# Nanorepairers Rescue Inflammation‐Induced Mitochondrial Dysfunction in Mesenchymal Stem Cells

**DOI:** 10.1002/advs.202103839

**Published:** 2021-12-11

**Authors:** Qiming Zhai, Xin Chen, Dongdong Fei, Xiaoyan Guo, Xiaoning He, Wanmin Zhao, Songtao Shi, John Justin Gooding, Fang Jin, Yan Jin, Bei Li

**Affiliations:** ^1^ State Key Laboratory of Military Stomatology & National Clinical Research Center for Oral Diseases & Shaanxi International Joint Research Center for Oral Diseases Center for Tissue Engineering School of Stomatology Fourth Military Medical University Xi'an Shaanxi 710032 China; ^2^ Department of Orthodontics School of Stomatology Fourth Military Medical University Xi'an Shaanxi 710032 China; ^3^ Department of Chemical Engineering Shaanxi Key Laboratory of Energy Chemical Process Intensification Institute of Polymer Science in Chemical Engineering School of Chemical Engineering and Technology Xi'an Jiao Tong University Xi'an Shaanxi 710049 China; ^4^ South China Center of Craniofacial Stem Cell Research Guanghua School of Stomatology Sun Yat‐sen University Guangzhou Guangdong 510080 China; ^5^ School of Chemistry and Australian Centre for Nano‐Medicine University of New South Wales Sydney NSW 2052 Australia

**Keywords:** chronic inflammation, mesenchymal stem cells, mitochondria dysfunction, nanoparticles

## Abstract

Mitochondrial dysfunction in tissue‐specific mesenchymal stem cells (MSCs) plays a critical role in cell fate and the morbidity of chronic inflammation‐associated bone diseases, such as periodontitis and osteoarthritis. However, there is still no effective method to cure chronic inflammation‐associated bone diseases by physiologically restoring the function of mitochondria and MSCs. Herein, it is first found that chronic inflammation leads to excess Ca^2+^ transfer from the endoplasmic reticulum to mitochondria, which causes mitochondrial calcium overload and further damage to mitochondria. Furthermore, damaged mitochondria continuously accumulate in MSCs due to the inhibition of mitophagy by activating the Wnt/*β*‐catenin pathway under chronic inflammatory conditions, impairing the differentiation of MSCs. Based on the mechanistic discovery, intracellular microenvironment (esterase and low pH)‐responsive nanoparticles are fabricated to capture Ca^2+^ around mitochondria in MSCs to regulate MSC mitochondrial calcium flux against mitochondrial dysfunction. Furthermore, the same nanoparticles are able to deliver siRNA to MSCs to inhibit the Wnt/*β*‐catenin pathway and regulate mitophagy of the originally dysfunctional mitochondria. These precision‐engineered nanoparticles, referred to as “nanorepairers,” physiologically restore the function of mitochondria and MSCs, resulting in effective therapy for periodontitis and osteoarthritis. The concept can potentially be expanded to the treatment of other diseases via mitochondrial quality control intervention.

## Graphical abstract

Chronic inflammation leads to mitochondrial Ca^2+^ overload and the inhibition of mitophagy, resulting in the impaired differention of mesenchymal stem cells (MSCs). Based on mechanistical findings, precision‐engineered nanoparticles physiologically restore the function of mitochondria and MSCs by capturing Ca^2+^ around mitochondria and inhibiting Wnt/*β*‐catenin pathway, providing effective therapy for periodontitis and osteoarthritis.

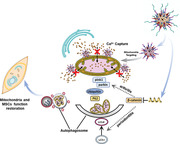

## 1 Introduction

Chronic inflammation is involved in various bone diseases, such periodontitis, arthritis, and osteoporosis.^[^ ^1^, ^2^, ^3^ ^]^ Inflammation impairs the proliferation, migration, and differentiation of tissue resident mesenchymal stem cells (MSCs, one of the most important cells in bone remodeling).^[^ ^4^, ^5^, ^6^ ^]^ The result can be serious bone damage that is unable to be regenerated by conventional approaches. Mitochondrial dysfunction in MSCs plays a critical role in damage associated with chronic inflammation.^[^ ^7^, ^8^, ^9^ ^]^ However, there is still no effective method to cure chronic inflammation‐associated bone diseases by physiologically restoring the function of the mitochondria in MSCs.

Recently, several studies have demonstrated that mitochondrial dysfunction originates from excess Ca^2+^ influx in obesity and Parkinson's disease.^[^ ^10^, ^11^ ^]^ Ca^2+^ overloads in mitochondria enhance the intracellular production of reactive oxygen species (ROS), which may damage relevant cells.^[^ ^8^, ^12^, ^13^ ^]^ Therefore, a few strategies targeting mitochondrial calcium channels by RNAi or peptides have been explored to reverse the mitochondrial dysfunction of cells.^[^ ^11^, ^14^ ^]^ Despite some unexpected clinical side effects, these studies inspired us to explore the critical role of excess Ca^2+^ influx into mitochondria underlying the impairment of MSCs in chronic inflammation and to develop an effective strategy for the regulation of mitochondrial Ca^2+^ in MSCs.

Dysfunctional mitochondria harm the function of cells due to impaired mitophagy,^[^ ^11^, ^14^, ^15^ ^]^ making the clearance of dysfunctional mitochondria even more important for treating chronic inflammation‐associated bone diseases. Current therapeutic strategies are only able to avoid the further deterioration of mitochondria caused by excess calcium, while they contribute little to removing the existing dysfunctional mitochondria.

**Figure 1:**
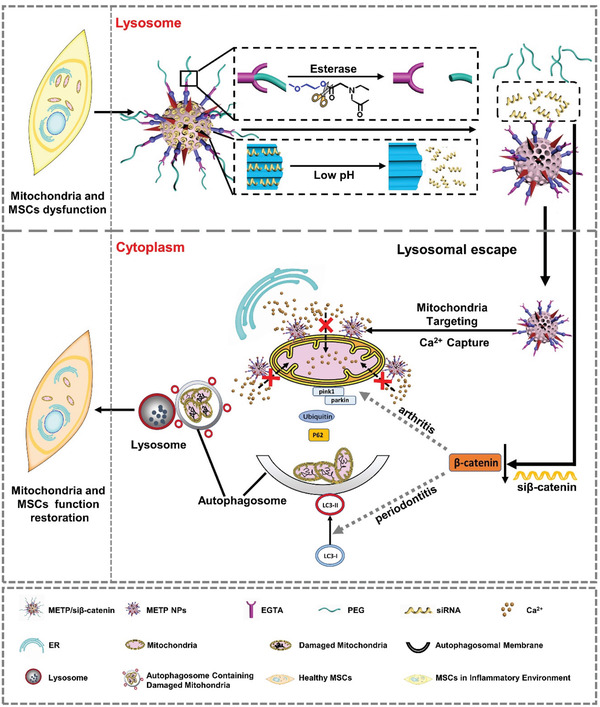
Schematic representation of the structure and function of METP NPs as well as how si*β*‐catenin loaded METP NPs (METP/si*β*‐catenin) sweep dysfunctional mitochondria and restore the function of mitochondria and MSCs. Chronic inflammation leads to excess Ca^2+^ transfer to mitochondria, which causes mitochondria calcium overload and the further damage of mitochondrial. Moreover, the damaged mitochondria continuously accumulate in MSCs due to the inhibition of mitophagy by the activation of Wnt/*β*‐catenin pathway under chronic inflammation condition (inhibiting the transfer from LC3II to LC3I for autophagosome formation in PDLSCs while decreasing the expression of pink1 and parkin to initiate mitophagy in BMMSCs), which impair the function of MSCs. Intracellular microenvironments (esterase and low pH)‐responsive nanoparticles are devised to capture Ca^2+^ around mitochondria in MSCs for regulating its mitochondria calcium flux against dysfunction of mitochondria, as well as to deliver si*β*‐catenin in MSCs to inhibit its Wnt/*β*‐catenin pathway for regulating mitophagy of dysfunctional mitochondria. The precision‐engineered nanoparticles, termed METP NPs, involve an amino functionalized mesoporous silica nanoparticles (MSN‐NH2) core as nanocarrier for si*β*‐catenin loading and pH triggered si*β*‐catenin release in targeted MSCs, an ethylene glycol tetraacetic acid (EGTA)/TPP composite shell as mitochondria‐targeted Ca^2+^ trapper, as well as a PEG corona connected with EGTA segments via ester bond. The ester bond would be cleaved by esterase to detach PEG corona after the endocytosis of MSCs, resulting in the activation of EGTA only in targeted MSCs, which was designed to avoid the disturbance of unexpected Ca^2+^ capture in the extracellular matrix.

Herein, we first found that chronic inflammation led to excess Ca^2+^ being partly transferred from the endoplasmic reticulum to the mitochondria. This Ca^2+^ transfer caused mitochondrial calcium overload and damage to the mitochondria. Furthermore, damaged mitochondria continuously accumulate in MSCs due to the inhibition of mitophagy by activating the Wnt/*β*‐catenin pathway under chronic inflammatory conditions, which then impairs the differentiation of MSCs (**Figure** **1**).

Our study attempts to explore an effective therapy based on the mechanistic findings. This approach requires a new strategy with the dual ability to specifically manipulate the levels of mitochondrial Ca^2+^ to inhibit the deterioration of healthy mitochondria and to eliminate the existing dysfunctional mitochondria for physiological restoration of mitochondrial quality control in MSCs. Therefore, we fabricated intracellular microenvironment (esterase and low pH)‐responsive nanoparticles to capture Ca^2+^ around the mitochondria in MSCs to manipulate mitochondrial calcium flux and to deliver siRNA in MSCs to inhibit the Wnt/*β*‐catenin pathway to regulate mitophagy of dysfunctional mitochondria. Precision‐engineered nanoparticles involve a positively charged mesoporous silica nanoparticle (TMA‐MSN) core as a nanocarrier for siRNA loading and pH‐triggered siRNA release in targeted MSCs, an ethylene glycol tetraacetic acid (EGTA, calcium chelator)/triphenylphosphine (TPP, mitochondrial targeting agent) composite shell to trap mitochondria‐related Ca^2+^, and a polyethylene glycol (PEG) corona connected with EDTA segments via ester bonds. These core–shell corona nanoparticles (TMA‐MSN‐EGTA/TPP‐PEG) were termed METP NPs. During therapy, the ester bond in METP NPs can be cleaved by esterase and low pH in lysosomes after the endocytosis of MSCs to detach the PEG corona, which not only activates the Ca^2+^ chelation ability of EGTA but also exposes TPP for mitochondrial binding. This leads to the in situ construction of Ca^2+^ trappers around mitochondria to block excess Ca^2+^ influx. The resulting Ca^2+^ regulation was utilized to rescue healthy mitochondria by avoiding excess Ca^2+^‐induced deterioration. Meanwhile, the lysosomal environment also triggers the release of preloaded RNA, which further inhibits the Wnt/*β*‐catenin pathway to eliminate dysfunctional mitochondria. These dual‐functional METP NPs were expected to be an effective therapy for several chronic inflammation‐associated bone diseases, including periodontal bone defects and osteoarthritis, which may further expand the treatment of other diseases via intervention of various impaired organelle functions.

## 2 Results

### 2.1 Ca^2+^ Overload in Mitochondria Results in Dysfunctional Mitochondria in MSCs Derived from Periodontitis and Osteoarthritis Patients

**Figure 2:**
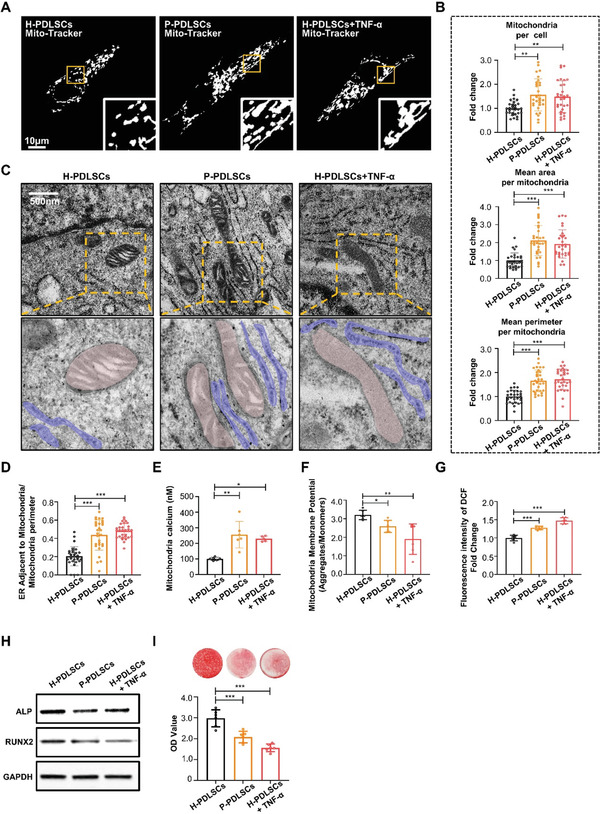
Mitochondrial Ca^2+^ overload results in dysfunctional mitochondria in MSCs derived from periodontitis and osteoarthritis patients. A) Representative images of H‐PDLSCs, P‐PDLSCs, and H‐PDLSCs+TNF‐*α* expressing Mito‐Tracker. Scale bar, 10 µm. B) Respective mitochondrial morphology analysis of cells by number of mitochondria per cell, and mean area and perimeter per mitochondrion (*n* = 30 cells in each group). C) Representative TEM of H‐PDLSCs, P‐PDLSCs, and H‐PDLSCs+TNF‐*α* at 43 000x, scale bar, 500 nm. Structures colored by purple indicate endoplasmic reticulum; Structures colored by pink indicate mitochondria. D) Quantitation of ER length adjacent to mitochondria normalized by mitochondrial perimeter (*n* = 30 cells in each group). E) Mitochondrial calcium was detected in H‐PDLSCs, P‐PDLSCs, and H‐PDLSCs+TNF‐*α* by Rhod‐2 (*n* = 6 independent samples). F) Mitochondrial membrane potential in H‐PDLSCs, P‐PDLSCs, and H‐PDLSCs+TNF‐*α* analyzed by JC‐1 assay (*n* = 6 independent experiments). G) ROS in H‐PDLSCs, P‐PDLSCs, and H‐PDLSCs+TNF‐*α* detected by DCFH‐DA assay (*n* = 6 independent experiments). H) The expression of osteogenesis‐associated protein in H‐PDLSCs, P‐PDLSCs, and H‐PDLSCs+TNF‐*α* was detected by western blot assay. Three experiments were repeated independently with similar results. I) Alizarin red staining showed that P‐PDLSCs and H‐PDLSCs+TNF‐*α* had a decreased capacity to form mineralized nodules when cultured under osteo‐inductive conditions compared to H‐PDLSCs (*n* = 6 independent samples in each group). *p < 0.05, **p < 0.01, ***p < 0.001.

We isolated tissue‐specific MSCs in the periodontal ligament, termed periodontal ligament stem cells (PDLSCs), from healthy (H‐PDLSCs) and periodontitis patients (P‐PDLSCs) (Figure S1A, Supporting Information). Tumor necrosis factor *α* (TNF‐*α*) was applied to treat H‐PDLSCs (H‐PDLSCs+TNF‐*α*) for 7 days to mimic a chronic inflammatory microenvironment in vitro. First, we characterized the morphology of the mitochondrial network by Mito‐Tracker in H‐PDLSCs, P‐PDLSCs, and H‐PDLSCs+TNF‐*α* (**Figure** **2**A). Analysis of the mitochondrial network revealed a greater number of mitochondria per cell in P‐PDLSCs and H‐PDLSCs+TNF‐*α* than in H‐PDLSCs (Figure 2B). Morphometric quantification of confocal microscopy images demonstrated that mitochondria in P‐PDLSCs and H‐PDLSCs+TNF‐*α* had a greater volume than H‐PDLSCs, as indicated by the mitochondrial perimeter and area (Figure 2A,B). Moreover, we observed that mitochondria were elongated in P‐PDLSCs and H‐PDLSCs+TNF‐*α*. This mitochondrial elongation was accompanied by a significantly increased ER and mitochondrial membrane apposition to each other, as determined by transmission electron microscopy (TEM) (Figure 2C). Detailed quantitative analysis of PDLSCs from each experimental group demonstrated that the proportion of ER in close contact with mitochondria was significantly higher in P‐PDLSCs and H‐PDLSCs+TNF‐*α* than in H‐PDLSCs (Figure 2D). We also utilized Mito‐Tracker (red) and ER‐Tracker (green) to specifically label these organelles and evaluated the abundance of mitochondrial‐ER contacts referred to as mitochondria‐associated membranes (MAMs)^[^ ^16^ ^]^ in H‐PDLSCs, P‐PDLSCs, and H‐PDLSCs+TNF‐*α* (Figure S2A, Supporting Information). Increased colocalization between the ER and mitochondria in P‐PDLSCs and H‐PDLSCs+TNF‐*α* was revealed by a significantly increased colocalization coefficient between the organelle‐targeted fluorescent markers (Figure S2B, Supporting Information). The abnormal mitochondria from P‐PDLSCs and H‐PDLSCs+TNF‐*α* contained significantly higher Ca^2+^ ([Ca^2+^]m) than those in H‐PDLSCs (Figure 2E).

The gene expression of specific proteins involved in the MAM subdomain, including MFN1, MFN2, IP3R1, IP3R2, IP3R3, GRP75, MCU, MICU1, and MICU2, was measured by reverse transcription polymerase chain reaction. The gene expression of MFN1 and MFN2, which is essential for the fusion of mitochondria, was upregulated in P‐PDLSCs and H‐PDLSCs+TNF‐*α*, suggesting mitochondrial hyperfusion under chronic inflammatory conditions (Figure S2C, Supporting Information). The gene expression level of the calcium channel IP3R2 was also significantly higher in P‐PDLSCs and H‐PDLSCs+TNF‐*α* than in H‐PDLSCs (Figure S2C, Supporting Information), indicating abnormal calcium flux into the mitochondria. We also observed a significant declined mitochondrial membrane potential (MMP) by JC‐1 assay and increased ROS production in P‐PDLSCss and H‐PDLSCs+TNF‐*α* compared to H‐PDLSCs by 2'‐7'dichlorofluorescin diacetate (DCFH‐DA) probe (Figure 2F,G). We performed measurements of oxygen consumption (OCR) and extracellular acidification rate (ECAR) to detect metabolism. The results showed that the maximum OCR, spare OCR, and ATP‐Coupled OCR were significantly lower in P‐PDLSCs than in H‐PDLSCs (Figure S2D, Supporting Information), as was the level of basal ECAR (Figure S2E, Supporting Information), indicating impaired mitochondrial function in P‐PDLSCs. In addition, the osteogenic‐related proteins alkaline phosphatase (ALP) and runt‐related transcription factor 2 (Runx2) were lower in P‐PDLSCs and H‐PDLSCs+TNF‐*α* (Figure 2H). The impaired osteogenic differentiation of P‐PDLSCs and H‐PDLSCs+TNF‐*α* was also revealed by decreased formation of calcium nodules, as assayed by Alizarin Red staining (Figure 2I). These results indicated that inflammation induces mitochondrial Ca^2+^ overload, leading to dysfunctional mitochondria and impaired function of MSCs.

To understand whether mitochondrial dysfunction is a universal phenomenon in MSCs derived from chronic inflammatory bone diseases, osteoarthritis was also used for further investigation. We isolated bone marrow MSCs (BMMSCs) from subchondral bone in arthritis patients (A‐BMMSCs), and BMMSCs cultured from the bone marrow of healthy donors (H‐BMMSCs) served as controls (Figure S1B, Supporting Information). TNF‐*α* was also applied to treat H‐BMMSCs (H‐BMMSCs+TNF‐*α*) for 7 days to mimic a chronic inflammatory microenvironment in vitro. The morphology of the mitochondrial network showed an increased number of individual mitochondria per cell, mitochondrial area and perimeter in A‐BMMSCs and H‐BMMSCs+TNF‐*α* compared to H‐BMMSCs (Figure S3A,B, Supporting Information). Increased mitochondrial volume was also observed in A‐BMMSCs and H‐BMMSCs+TNF‐*α* (Figure S3C, Supporting Information). Intriguingly, again these swollen mitochondria exhibited greater contact with the ER in A‐BMMSCs and H‐BMMSCs+TNF‐*α* (Figure S3C,D, Supporting Information). In addition, the mitochondria from A‐BMMSCs and H‐BMMSCs+TNF‐*α* also contained significantly higher Ca^2+^ ([Ca^2+^]m) than H‐BMMSCs (Figure S3E, Supporting Information). We also observed a significantly decreased mitochondrial membrane potential (MMP) and increased ROS production in A‐BMMSCs and H‐BMMSCs+TNF‐*α* compared to H‐BMMSCs (Figure S3F,G, Supporting Information), indicating impaired mitochondrial function.

### 2.2 Obstructed Mitophagy Leads to the Accumulation of Dysfunctional Mitochondria in MSCs Derived from Periodontitis and Osteoarthritis Patients

**Figure 3:**
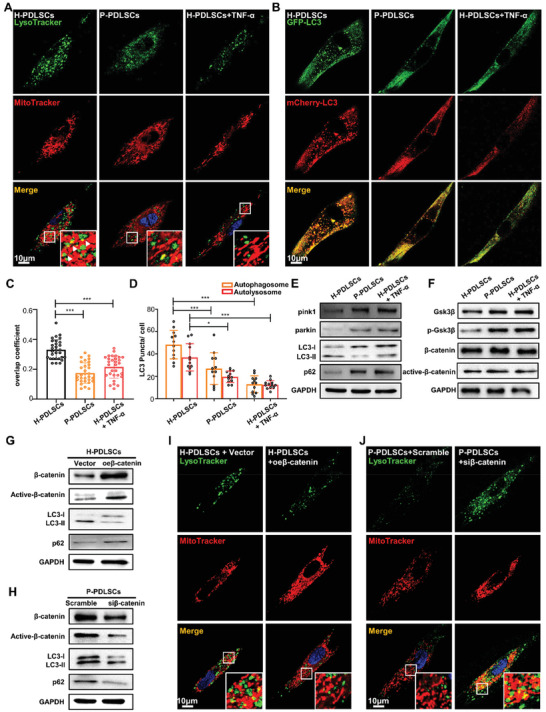
Obstructed mitophagy leads to accumulation of the dysfunctional mitochondria in MSCs derived from periodontitis patients. A) Representative confocal microscopy images of H‐PDLSCs, P‐PDLSCs, and H‐PDLSCs+TNF‐*α* co‐expressing a Mito‐Tracker (red) and Lyso‐Tracker (green). Scale bar, 10 µm. B) Representative confocal microscopy images of H‐PDLSCs, P‐PDLSCs, and H‐PDLSCs+TNF‐*α* transfected with the tandem mRFP‐GFP‐LC3 plasmids. Scale bar, 10 µm. C) Statistical quantification of the overlap coefficient between Mito‐Tracker (red) and Lyso‐Tracker (green) (*n* = 30 cells in each group). D) The numbers of yellow LC3 dots and red LC3 dots per cell in each condition were quantified (*n* = 12 cells in each group). E) The expression of pink1, parkin, LC3I, LC3II, and p62 in H‐PDLSCs, P‐PDLSCs, and H‐PDLSCs+TNF‐*α* was detected by western blot assay. F) The expression of GSK3*β*, pGSK3*β*, *β*‐catenin, and active *β*‐catenin in H‐PDLSCs, P‐PDLSCs, and H‐PDLSCs+TNF‐*α* was detected by western blot assay. G) The expression of *β*‐catenin, active *β*‐catenin, LC3I, LC3II, and p62 was measured in H‐PDLSCs and H‐PDLSCs overexpressed with *β*‐catenin. H) The expression of *β*‐catenin, active *β*‐catenin, LC3I, LC3II, and p62 were measured in P‐PDLSCs and P‐PDLSCs transfected with si*β*‐catenin. I) Representative confocal microscopy images of H‐PDLSCs and H‐PDLSCs overexpressed with *β*‐catenin co‐expressing a Mito‐Tracker (red) and Lyso‐Tracker (green). Scale bar, 10 µm. J) Representative confocal microscopy images of P‐PDLSCs and P‐PDLSCs transfected with si*β*‐catenin co‐expressing Mito‐Tracker (red) and Lyso‐Tracker (green). Scale bar, 10 µm. **p < 0.01, ***p < 0.001.

As an increased number of elongated mitochondria in MSCs under chronic inflammatory conditions was observed (Figure 2A and Figure S3A, Supporting Information), we hypothesized that dysfunctional mitochondria were not eliminated by mitophagy, which selectively removes damaged or excessive mitochondria.^[^ ^15^ ^]^ To verify whether mitophagy was inhibited after mitochondrial damage, we stained the mitochondria and lysosomes of PDLSCs with Mito‐Tracker (red) and Lyso‐Tracker (green), respectively. Compared with H‐PDLSCs, colocalization of mitochondria and lysosomes was significantly lower in the P‐PDLSCs and H‐PDLSCs+TNF‐*α* (**Figure** **3**A,C). This lower colocalization indicates the deficiency of mitophagy in these cells. Apart from using colocalization estimation of Mito‐Tracker (red) and Lyso‐Tracker (green), we also transfected PDLSCs with GFP‐RFP‐LC3 lentivirus. This probe can be used to identify autophagosomes (GFP positive/RFP positive; yellow dots) and autolysosomes (GFP‐negative/RFP‐positive; red dots) because GFP fluorescence is quantitatively quenched in low pH compartments.^[^ ^17^ ^]^ Analysis of the distribution of LC3 puncta showed that the number of both yellow and red puncta decreased in P‐PDLSCs and H‐PDLSCs+TNF‐*α*, indicating that autophagic flux was suppressed under chronic inflammatory conditions (Figure 3B,D). Moreover, pink1 and parkin, which accumulate in dysfunctional mitochondria and initiate mitophagy, were increased significantly in P‐PDLSCs and H‐PDLSCs+TNF‐*α*, as determined by western blot, indicating that dysfunctional mitochondria were ready to be eliminated by mitophagy (Figure 3E). However, LC3II/I was decreased while p62 was increased in the inflammatory groups (Figure 3E), demonstrating that the formation of autophagosomes was suppressed and that labeled dysfunctional mitochondria were further inhibited from being sent to lysosomes for degradation.

The Wnt/*β*‐catenin signaling pathway is vital in influencing the fate of MSCs under chronic inflammatory conditions, as well as in regulating mitochondrial function.^[^ ^18^, ^19^ ^]^ The Wnt/*β*‐catenin pathway was activated in P‐PDLSCs and H‐PDLSCs+TNF‐*α*, as evident from the increased expression of p‐GSK3*β* and active *β*‐catenin (Figure 3F). We also found that overexpression of *β*‐catenin in H‐PDLSCs suppressed LC3‐I transformation to LC3‐II (Figure 3G) and inhibited the formation of autophagosomes and autolysosomes in H‐PDLSCs (Figure S4A, Supporting Information). In contrast, impaired LC3 transformation (Figure 3H) and autophagosome (yellow)/autolysosome (red) generation (Figure S4B, Supporting Information) could be rescued after knockdown of *β*‐catenin in P‐PDLSCs. Additionally, the colocalization of mitochondria and lysosomes in H‐PDLSCs was decreased after *β*‐catenin overexpression (Figure 3I), while elevated colocalization of Mito‐Tracker and Lyso‐Tracker in P‐PDLSCs was observed after knockdown of *β*‐catenin with siRNA (Figure 3J).

In addition, we also found inhibited mitophagy in BMMSCs under chronic inflammation due to the activation of the Wnt/*β*‐catenin signaling pathway (Figure S5, Supporting Information). The Wnt/*β*‐catenin pathway was activated in A‐BMMSCs and H‐BMMSCs+TNF‐*α*, as marked by increased expression of p‐GSK3*β* and active‐*β*‐catenin (Figure S5A, Supporting Information). However, lower pink1 and parkin levels were found in A‐BMMSCs and H‐BMMSCs+TNF‐*α*, as determined using western blots (Figure S5B, Supporting Information). After overexpression of *β*‐catenin in H‐BMMSCs, the expression of pink1 and parkin was decreased, indicating that dysfunctional mitochondria might be inhibited in the initiation of mitophagy in BMMSCs under inflammation. Taken together, these results demonstrated that activation of the Wnt/*β*‐catenin pathway impaired autophagosome generation and led to deficiency of mitophagy in MSCs from chronic inflammatory bone diseases. Therefore, the accumulation of dysfunctional mitochondria contributed to the dysfunction of MSCs.

### 2.3 Precise Fabrication of RNA‐Loaded METP NPs to Regulate Mitochondrial Calcium and Mitophagy in MSCs

**Figure 4:**
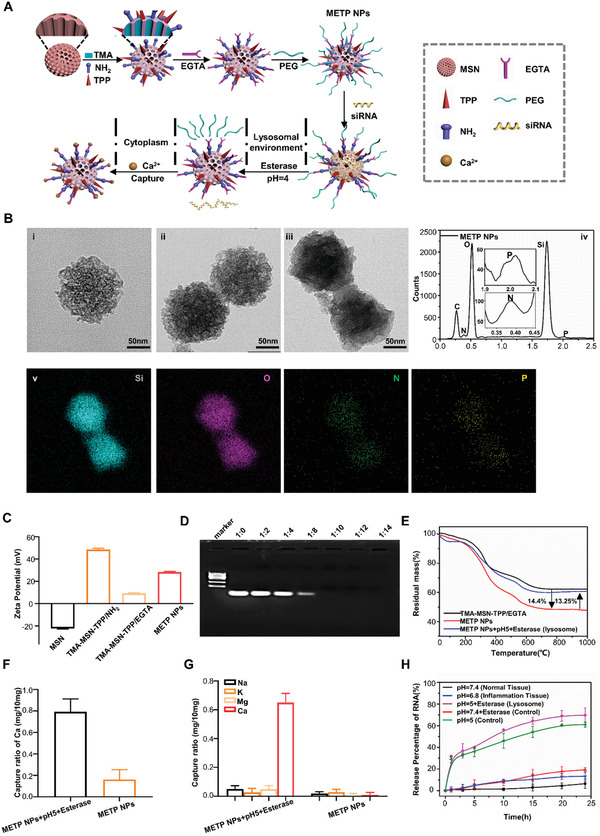
Precise fabrication of METP NPs to regulate mitochondrial calcium and mitophagy in MSCs. A) Engineering METP NPs to regulate ER‐mitochondria calcium and mitophagy precisely. B) Transmission electron microscope (TEM) images of i) MSN, ii) TMA‐MSN‐TPP/EGTA, iii) TMA‐MSN‐TPP/EGTA‐PEG (METP NPs) as well as the corresponding EDS analysis of METP NPs in iv) spectrum and v) mapping model. C) Surface charge of MSN, TMA‐MSN‐TPP/NH_2_, TMA‐MSN‐TPP/EGTA, and METP NPs determined by Zeta potential measurement. D) Polyacrylamide gel electrophoresis of METP NPs after incubation with different amounts of RNA. E) Thermogravimetric (TG) curves of TMA‐MSN‐TPP/EGTA as well as METP NPs before and after esterase treatment at pH 5.0 (simulated lysosome condition) to determine the PEG cleavage in lysosome. F,G) The Ca^2+^ capture ability of METP NPs before and after esterase/acid treatment in F) pure Ca^2+^ solutions as well as G) in mixed solutions containing Na^+^, K^+^, Mg^2+^, and Ca^2+^ determined by high‐performance ion chromatograph (HPIC). H) Release percentage of RNA from METP NPs under different simulated physiological conditions.

According to the mechanistic findings of mitochondria‐related dysfunction of PDLSCs in periodontitis and BMMSCs in osteoarthritis, we engineered METP NPs to regulate mitochondrial calcium and mitophagy precisely for effective therapy of periodontitis and osteoarthritis. The nanoparticles were modified by EGTA, which is able to conjugate with Ca^2+^ via coordinate bond to generate stable metal complex with high complexation constant at 10.97, resulting in the effective capture of Ca^2+^. To prepare METP NPs, mesoporous silica nanoparticles (MSNs) were first synthesized with amino groups and TPP (mitochondrial targeting agent) on the external surface and trimethyl [3‐(trimethoxysilyl) propyl] ammonium chloride (TMA, RNA binding agent) in the pores. The amino groups were employed to introduce ethylenebis (oxyethylenenitrilo) tetraacetic acid (EGTA, Ca^2+^ chelator) via amidation. The residual carboxyl groups on EGTA were further conjugated with PEG chains via an esterification reaction to produce the final METP NPs (**Figure** **4**A).

The stepwise construction of METP NPs was first characterized using TEM and energy dispersive X‐ray spectroscopy (EDS). Figure 4B shows that the as‐synthesized MSNs and TMA, TPP, and EGTA co‐modified MSNs (TMA‐MSN‐TPP/EGTA) all have a spherical structure with a diameter of ≈150 nm (i and ii). An obvious organic layer and a clear size increase were evident after PEG grafting, indicating the formation of METP NPs (iii). Further information was provided by the EDS (iv and v) and infrared (IR) spectrometer (Figure S6A, Supporting Information) of the METP NPs, which revealed characteristic signals of the various elements and functional groups expected. The stepwise modification also caused dramatic changes in the surface charge, which is consistent with the electrostatic properties of these functional groups, ending with a positive charge of ≈30 mV (METP NPs) for the future encapsulation of RNA (Figure 4C). The loading capacity of RNA in METP NPs was investigated by polyacrylamide gel electrophoresis, which indicated complete RNA encapsulation by the nanoregulator once the mass ratio between RNA and METP NPs reached 1:10 (Figure 4D). The surface charge of METP NPs after RNA loading (1:10) was also estimated by the zeta potential and remained slightly positive (Figure S6B, Supporting Information), which is important to ensure effective intracellular delivery of the therapeutic RNA. Subsequent experiments were performed with this ratio.

The RNA‐loaded METP NPs were mostly taken up by target cells via endocytosis, which inevitably passed through lysosomes containing abundant esterase and acidic environments.^[^ ^20^, ^21^ ^]^ The Ca^2+^ capture and RNA release abilities of the METP NPs were designed to be triggered by esterase and a low pH value. As shown in Figure 4E, over 90% of the PEG chains on the surface of METP NPs were removed after esterase treatment at pH 5.0 due to the enzymolysis and hydrolysis of ester bonds, which exposed EGTA to capture the pathologically overexpressed Ca^2+^ around mitochondria after lysosome escape and TPP‐induced mitochondrial targeting. The esterase‐dependent Ca^2+^ capture of METP NPs was quantitatively investigated by ion chromatography, which indicated a high Ca^2+^ trapping percentage only after esterase treatment at pH 5.0 either in the solution containing only Ca^2+^ (Figure 4F) or in the mixed solution containing Ca^2+^, Mg^2+^, Na^+^, and K^+^ (major ions in cells, Figure 4G). These results indicated that the METP NPs could perform precise Ca^2+^ capture in and only in target cells to effectively downregulate pathological Ca^2+^, and the other ions in cells did not affect the therapeutic efficiency of these METP NPs.

The pH‐dependent release behavior of RNA from the METP NPs was investigated by fluorescence spectroscopy using fluorescein isothiocyanate (FITC)‐labeled RNA under various simulated physiological conditions. The results showed that the RNA only escaped from METP NPs in the simulated acidic environment of lysosomes (pH 5.0), ending with a cumulative release percentage of ≈70% in 24 h due to the protonation process (Figure 4H). This value could be further enhanced by extra treatment with esterase (rich in lysosomes) due to the accelerated detachment of PEG chains, which previously limited the spread of RNA. These results indicated that the METP NPs could perform effective RNA delivery and subsequent intracellular release in target cells for designed gene therapy.

Before therapy, the cytotoxicity of METP NPs with and without RNA loading was evaluated. We did not find an inhibition of cell proliferation in PDLSCs and BMMSCs after treatment with METP NPs, si*β*‐catenin‐loaded TMA‐MSNs‐TPP (TMA‐MSNs‐TPP/si*β*‐catenin, control group without capacity of Ca^2+^ capture), and si*β*‐catenin‐loaded METP NPs (METP/si*β*‐catenin NPs, Figure S6C,D, Supporting Information).

### 2.4 METP/si*β*‐Catenin Restores Mitochondrial Function of Diseased MSCs and Rescues Periodontal Bone Loss

**Figure 5:**
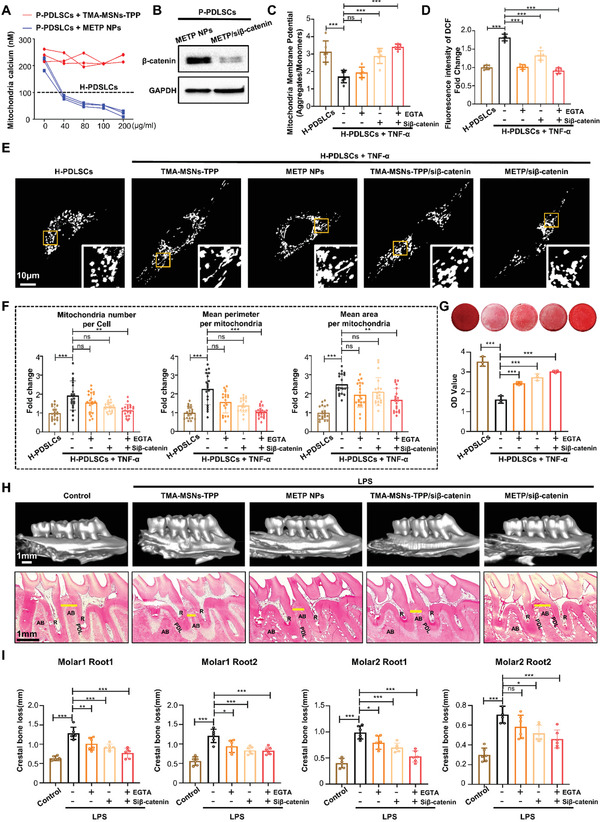
METP/si*β*‐catenin restores mitochondrial function of diseased MSCs and rescues periodontal bone loss. A) Mitochondrial calcium was detected in P‐PDLSCs with treatment of TMA‐MSNs‐TPP and METP NPs with concentrations of 0, 40, 80, 100, and 200 µg mL^−1^ (*n* = 3 independent experiments). B) The expression of *β*‐catenin in P‐PDLSCs with treatment of METP NPs and METP/si*β*‐catenin was detected by western blot assay. C) Recovery of mitochondrial membrane potential in PDLSCs with treatment of TNF‐*α* and different NPs (*n* = 6 independent experiments). D) Recovery of ROS levels in PDLSCs with treatment of TNF‐*α* and different NPs (*n* = 6 independent experiments). E) Representative images of H‐PDLSCs and H‐PDLSCs+TNF‐*α* with treatment of TMA‐MSNs‐TPP, METP NPs, TMA‐MSNs‐TPP/si*β*‐catenin, METP/si*β*‐catenin expressing Mito‐Tracker. Scale bar, 10 µm. F) Respective mitochondrial morphology analysis of cells by number of mitochondria per cell, and mean area and perimeter per mitochondrion (*n* = 20 cells in each group). G) Alizarin red staining showed that H‐PDLSCs+TNF‐*α* with treatment of METP NPs, TMA‐MSNs‐TPP/si*β*‐catenin, and METP/si*β*‐catenin had an increased capacity to form mineralized nodules when cultured under osteo‐inductive conditions compared to treatment of TMA‐MSNs‐TPP (*n* = 6 independent experiments). H) The alveolar bone loss of control and LPS‐induced periodontitis SD rats (*n* = 6 rats for each group) was determined by micro‐CT and H&E staining. Scale bar, 1 mm. P: pulp; D: dentine; PDL: periodontal ligament; AB: alveolar bone. I) Four sites for two molars (one site for each root of one tooth) were analyzed morphometrically. The results of micro‐CT and H&E staining showed METP/si*β*‐catenin treatment rescued alveolar bone loss in periodontitis SD rats compared to the TMA‐MSNs‐TPP, METP NPs, and TMA‐MSNs‐TPP/si*β*‐catenin groups (*n* = 6 independent samples). *p < 0.05, **p < 0.01, ***p < 0.001, ns, not significant.

The mitochondrial targeting effect of METP NPs was verified by confocal fluorescence microscopy after 2 h of incubation with PDLSCs (Figure S6E, Supporting Information), in which the METP NPs were labeled with FITC (green) and the PDLSCs were stained with Mito‐Tracker (red). The mitochondrial calcium content after treatment with METP NPs was further measured to evaluate the efficacy of mitochondrial calcium flux blockade, which was significantly decreased in P‐PDLSCs after METP NP treatment for 3 days (**Figure** **5**A). Notably, the mitochondrial calcium content in P‐PDLSCs treated with 40 µg mL^−1^ METP NPs decreased to ≈100 × 10^−9^ m, which is the same as the physiological mitochondrial calcium level in H‐PDLSCs. Thus, 40 µg mL^−1^ METP NPs was used in the following experiments. After 3 days of treatment with METP NPs loaded with si*β*‐catenin, the expression of *β*‐catenin in P‐PDLSCs decreased, as determined by western blot assays (Figure 5B).

To validate whether the si*β*‐catenin‐loaded METP NPs could reverse the disordered mitochondrial function and the impaired osteogenic differentiation potential of PDLSCs in inflammation, H‐PDLSCs+TNF‐*α* were cultured with TMA‐MSNs‐TPP (nanoparticles without the capacity of Ca^2+^ capture and si*β*‐catenin), METP NPs (nanoparticles with the capacity of Ca^2+^ capture only), TMA‐MSNs‐TPP/si*β*‐catenin (nanoparticles with the capacity of si*β*‐catenin only), and METP/si*β*‐catenin (nanoparticles with the capacity of both mitochondrial Ca^2+^ capture and si*β*‐catenin) for 3 days. METP/si*β*‐catenin showed the best effect in restoring the function of mitochondria, as measured by mitochondrial membrane potential (Figure 5C) and ROS levels (Figure 5D). Systematic confocal microscopy analysis of the mitochondrial network revealed a decrease in the number of mitochondria, mitochondrial area, and perimeter in METP NPs‐, TMA‐MSNs‐TPP/si*β*‐catenin‐, and METP/si*β*‐catenin‐treated H‐PDLSCs+TNF‐*α* compared to H‐PDLSCs+TNF‐*α* treated with TMA‐MSNs‐TPP (Figure 5E,F). Furthermore, the proportion of MAMs was significantly higher in TMA‐MSNs‐TPP‐treated than in METP/si*β*‐catenin‐treated H‐PDLSCs+TNF‐*α*, relative to the mitochondrial perimeter (Figure S7A, Supporting Information). Meanwhile, the decreased maximal OCR (Figure S7B, Supporting Information) and basal ECAR (Figure S7C, Supporting Information) in P‐PDLSCs can be reversed by METP/si*β*‐catenin NPs. Mineralization nodule formation by Alizarin red staining showed recovered osteogenic differentiation of METP NPs‐, TMA‐MSNs‐TPP/si*β*‐catenin‐, and METP/si*β*‐catenin‐treated H‐PDLSCs+TNF‐*α* (Figure 5G). The osteogenic‐related proteins ALP and Runx2 detected by western blotting also showed the enhanced osteogenic differentiation of METP NPs, TMA‐MSNs‐TPP/si*β*‐catenin‐, and METP/si*β*‐catenin‐treated H‐PDLSCs+TNF‐*α* (Figure S7D, Supporting Information). Intriguingly, METP/si*β*‐catenin appeared to be more efficient in rescuing mitochondrial function and osteogenic differentiation than METP NPs and TMA‐MSNs‐TPP/si*β*‐catenin (Figure 5C–G and Figure S7D, Supporting Information).

Subsequently, to evaluate the effect of METP NPs on preventing alveolar bone loss in lipopolysaccharide (LPS)‐induced periodontitis rats, TMA‐MSNs‐TPP, METP NPs, TMA‐MSNs‐TPP/si*β*‐catenin, and METP/si*β*‐catenin were injected at the same sites between the first and second molars in periodontitis rats. The rats without LPS injection were used as controls. Micro‐CT examination and hematoxylin and eosin (H&E) staining were applied to quantify alveolar bone loss in periodontal tissues after 28 days of NP treatment. The alveolar bone treated with LPS and empty TMA‐MSNs‐TPP was severely lost, while the same bone loss was not observed after treatment with METP NPs, TMA‐MSNs‐TPP/si*β*‐catenin, and METP/si*β*‐catenin (Figure 5H,I). Furthermore, METP/si*β*‐catenin treatment showed significantly more regenerated bone than METP NPs and TMA‐MSNs‐TPP/si*β*‐catenin groups, as the distance from the cementoenamel junction (CEJ) to the alveolar bone crest after METP/si*β*‐catenin treatment was similar to that of the normal control group (Figure 5I). Taken together, dysfunctional mitochondria caused by increased mitochondrial calcium and obstructed mitophagy led to impaired osteogenic differentiation of PDLSCs. METP/si*β*‐catenin functionalized with EGTA and si*β*‐catenin showed the best therapeutic effect on rescuing the function of mitochondria and PDLSCs, which provided a therapeutic effect on alveolar bone loss in periodontitis.

### 2.5 METP/si*β*‐Catenin Restores Mitochondrial Function of Diseased MSCs and Alleviates Osteoarthritis

**Figure 6:**
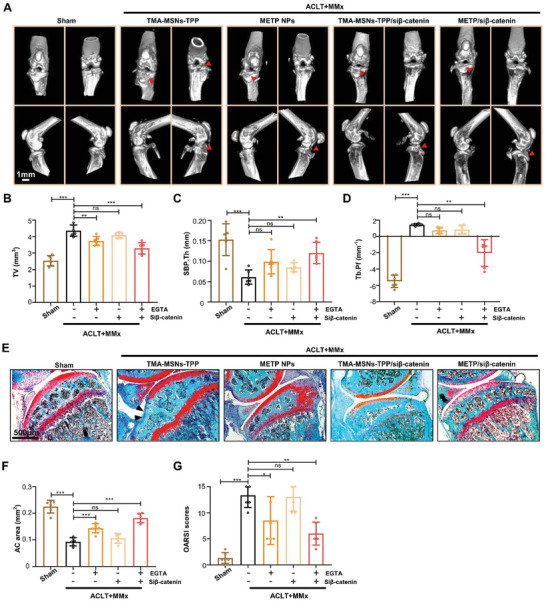
METP/si*β*‐catenin restores mitochondrial function of diseased MSCs and alleviates osteoarthritis. A) Representative micro‐CT images of ACLT+MMx mice after treated with TMA‐MSNs‐TPP, METP NPs, TMA‐MSNs‐TPP/si*β*‐catenin, METP/si*β*‐catenin, or sham surgery (*n* = 6 mice for each group). Red arrowheads indicate destructions at tibial subchondral bone. Scale bar, 1 mm. B–D) Quantitative analysis of B) total tissue volume (TV), C) thickness of SBPs (SBP Th), and D) Tb. Pf in subchondral bone determined by micro‐CT analysis (*n* = 6 independent samples). E) Safranin O and fast green staining of sagittal sections of the tibia medial compartment, proteoglycan (red), and bone (blue). Black arrowheads indicate loss of cartilage. Scale bar, 500 µm. F) Quantification of AC area (*n* = 6 independent samples). G) Osteoarthritis research society international (OARSI) scoring system was used to evaluate knee joint articular cartilage destruction (*n* = 6 independent samples). *p < 0.05, **p < 0.01, ***p < 0.001, ns, not significant.

To further show that METP/si*β*‐catenin rescued dysfunctional mitochondria in A‐BMMSCs derived from osteoarthritis patients, mitochondrial membrane potential and ROS production were measured by JC‐1 assay and DCFH‐DA probe, respectively. METP/si*β*‐catenin treatment showed the best effect in restoring the function of mitochondria compared to the TMA‐MSNs‐TPP, METP NPs, and TMA‐MSNs‐TPP/si*β*‐catenin groups (Figure S7E,F, Supporting Information). However, the osteogenesis‐related proteins (ALP and Runx2) of H‐BMMSCs+TNF‐*α* were increased compared to those of H‐BMMSCs and were significantly downregulated by METP/si*β*‐catenin treatment (Figure S7G, Supporting Information). Furthermore, to evaluate the effect of different functionalized NPs on the prevention of joint destruction in OA mice (anterior cruciate ligament transection and partial medial meniscectomy in 8 week old C57BL/6 mice, ACLT+MMx mice), TMA‐MSNs‐TPP, METP NPs, TMA‐MSNs‐TPP/si*β*‐catenin, and METP/si*β*‐catenin were injected into subchondral bone in OA mice. Strikingly, destruction on the surface of joints accompanied by ectopic bone spurs and osteophytes in the OA mice was observed in 3D micro‐CT reconstruction images (**Figure** **6**A). The total subchondral bone tissue volume (TV) was increased, while the thickness of the subchondral bone plate (SBP) was decreased in ACLT+MMx mice compared to sham‐operated controls by 2 months after surgery (Figure 6B,C). Moreover, a significant increase in the trabecular pattern factor (Tb. Pf) in the ACLT+MMx mice indicated uncoupled bone remodeling and the disruption of microarchitecture in trabecular bone (Figure 6D). These disorders could be recovered by METP NPs, TMA‐MSNs‐TPP/si*β*‐catenin, and METP/si*β*‐catenin to varying degrees. Moreover, METP/si*β*‐catenin treatment showed significantly better therapeutic effects than METP NPs and TMA‐MSNs‐TPP/si*β*‐catenin (Figure 6A–D).

Additionally, highly abrasive articular cartilage (AC) in ACLT+MMx mice treated with TMA‐MSNs‐TPP was observed by safranin O and fast green staining. Meanwhile, the integrity and continuity of AC was rescued by METP NP, TMA‐MSN‐TPP/si*β*‐catenin, and METP/si*β*‐catenin treatments 2 months after surgery (Figure 6E,F), a time point that is often used for analysis in destabilized OA mouse models.^[^ ^22^ ^]^ Osteoarthritis Research Society International (OARSI) scores revealed degeneration of articular cartilage after ACLT+MMx and significant recovery after METP NPs and METP/si*β*‐catenin treatment (Figure 6G). The best effect of METP/si*β*‐catenin treatment on articular cartilage was quantified (Figure 6G). Taken together, METP/si*β*‐catenin showed the best therapeutic effect on remodeling the network of mitochondria in BMMSCs from OA and finally rescued the disruption in microarchitecture of knee joints.

## 3 Discussion

In this study, we first found that due to increased mitochondrial calcium and obstructed mitophagy, excess dysfunctional mitochondria caused disordered differentiation of MSCs derived from inflammatory bone diseases. To restore the function of mitochondria and MSCs, we fabricated mitochondria‐targeting and intracellular microenvironment (esterase and low pH)‐responsive nanoparticles termed METP to capture Ca^2+^ around mitochondria in MSCs to regulate calcium flux into mitochondria against mitochondrial dysfunction and to deliver siRNA (si*β*‐catenin) to MSCs to inhibit the Wnt/*β*‐catenin pathway to regulate mitophagy and remove the dysfunctional mitochondria. The specific responsiveness and selectivity of METP NPs, which we called a “nanorepairers,” allows all of the activities to occur only in diseased MSCs to achieve effective recovery of mitochondria without off‐target effects, resulting in significant alleviation of periodontitis and osteoarthritis. As such, this work provides a promising strategy to treat various chronic inflammation‐associated bone diseases.

Decreased osteogenic differentiation of PDLSCs, which are derived from the neural crest, is considered the most critical cause of periodontitis.^[^ ^5^, ^6^ ^]^ However, increased osteogenic differentiation of BMMSCs in subchondral bone, which is derived from mesoderm, has also been reported as an important reason for osteoarthritis.^[^ ^4^, ^23^ ^]^ Chronic inflammatory conditions led to organelle dysfunction of tissue‐specific PDLSCs and BMMSCs, such as endoplasmic reticulum stress,^[^ ^5^ ^]^ activation of autophagy,^[^ ^24^ ^]^ and increased mitochondrial ROS,^[^ ^25^ ^]^ which induces impaired proliferation or differentiation of these cells related to the symptoms of periodontitis and osteoarthritis.

Excessive Ca^2+^ influx into mitochondria causes damage with compromised mitochondrial oxidative capacity and augmented oxidative stress,^[^ ^10^ ^]^ resulting in important consequences for the fate of MSCs.^[^ ^26^ ^]^ This dysfunction in MSCs is closely related to inflammation‐related bone diseases, such as periodontitis and osteoarthritis. Here, we first found significant mitochondrial Ca^2+^ overload in PDLSCs from periodontitis patients and BMMSCs from OA patients, which impaired mitochondrial function, including decreased mitochondrial membrane potential and increased ROS production, resulting in abnormal differentiation of the cells. Dysfunctional mitochondria should be removed by mitophagy, the selective degradation of mitochondria by autophagy, to prevent cell damage and death.^[^ ^27^, ^28^, ^29^ ^]^ However, these damaged mitochondria could not be eliminated, as assayed by mitochondrial mass quantification in PDLSCs and BMMSCs from patients. Furthermore, we found that the Wnt/*β*‐catenin pathway was activated to inhibit the mitophagy process, leading to an increased number of mitochondria. The Wnt/*β*‐catenin pathway inhibited the transfer from LC3II to LC3I for autophagosome formation in PDLSCs, while it decreased the expression of pink1 and parkin to initiate mitophagy in BMMSCs, as described in Figure 1. Therefore, there is a need to devise novel approaches to both regulate mitochondrial calcium and remove damaged mitochondria by activating mitophagy in MSCs from chronic inflammation‐associated bone diseases.

Recently, small molecule pharmacological tools have shown considerable value for dissecting complex mitochondrial biological processes and identifying potential therapeutic interventions. To date, several peptides targeting the main Ca^2+^‐release channels on the ER and mitochondria have been found to regulate mitochondrial calcium influx.^[^ ^14^ ^]^ However, the pleiotropy of many targets and the resultant effects on cell function could lead to various unexpected clinical effects, limiting the use of these peptides. Compared with the approaches of reducing the Ca^2+^ concentration in MSCs by targeting the main Ca^2+^‐release channels on the ER and mitochondria by peptides, our METP NPs can precisely cut off excessive calcium flux to mitochondria as needed, not only reversing mitochondrial dysfunction but also avoiding the unexpected clinical effects caused by weak affinity and low potency.^[^ ^30^ ^]^

To restore the ability of cells to efficiently eliminate dysfunctional mitochondria, current approaches to initiate mitophagy include acute dissipation of the mitochondrial membrane potential by mitochondrial uncouplers (e.g., FCCP/CCCP) and the use of antimycin A and oligomycin to impair respiration.^[^ ^31^ ^]^ To avoid off‐target effects of these chemicals, our strategy employs siRNA to replace toxic chemicals to promote the mitophagy of dysfunctional mitochondria, which not only establishes a new therapeutic method by regulating the Wnt/*β*‐catenin pathway but also prevents possible cell damage caused by the chemicals themselves and the accompanying acute depolarization of mitochondria.^[^ ^32^ ^]^ The in vitro results also indicated that METP/si*β*‐catenin could downregulate mitochondrial calcium and increase mitophagy in both PDLSCs and BMMSCs, thus restoring the function of diseased MSCs. MSCs are thought to exist in joint tissues and play important roles in joint homeostasis and maintenance.^[^ ^33^, ^34^ ^]^ Importantly, MSCs secrete large amounts of growth factors and immunomodulatory factors to suppress inflammation and promote repair of cartilage.^[^ ^35^, ^36^ ^]^ Therefore, the injection of METP/si*β*‐catenin restores mitochondrial function of diseased MSCs and further reverts the cartilage phenotype.

Intriguingly, METP/si*β*‐catenin was also able to restore the tethering between mitochondria and endoplasmic reticulum (ER) membranes, indicating that the balance of calcium flux to mitochondria reconstructs the structure of the organelles. However, we did not explore the relationship of mitochondrial calcium and mitophagy in this study, as a previous study showed that mitochondrial calcium homeostasis is critical for pink1's function in mitophagy priming.^[^ ^11^ ^]^

## 4 Conclusion

In conclusion, our study revealed dysfunctional mitochondria were caused by excessive calcium to mitochondria and accumulated damaged mitochondria due to inhibited mitophagy in MSCs derived from chronic inflammatory bone diseases. Based on our mechanistic findings, we devised novel “mitochondrial nanorepairers,” mitochondria‐targeting and intracellular microenvironment (esterase and low pH)‐responsive nanoparticles, to capture Ca^2+^ around mitochondria in MSCs to regulate mitochondrial calcium flux against mitochondrial dysfunction and to deliver siRNA to MSCs to inhibit the Wnt/*β*‐catenin pathway to regulate mitophagy and remove damaged mitochondria. This mitochondrial calcium calibrator showed therapeutic effects on periodontitis and osteoarthritis, providing a promising strategy to treat various chronic inflammation‐associated bone diseases.

## 5 Experimental Section

### Chemicals

NH_3_·H_2_O (AR, 25–28%) was purchased from Xi'an Sanpu Chemical Reagent Co., Ltd. (Xi'an, China). Aminopropyltriethoxysilane (APTES) was purchased from Energy Chemical (Shanghai, China). Ethylenebis(oxyethylenenitrilo)tetraacetic acid (EGTA), trimethyl[3‐(trimethoxysilyl)propyl]ammonium chloride (≈50% methanol), TPP, *N*,*N*‐dicyclohexyl carbonate diimide (DCC), 4‐dimethylaminopyridine (DMAP), 1‐(3‐dimethylaminopropyl)‐3‐ethylcarbodiimide hydro (EDC), *N*‐hydroxysuccinimide (NHS), and 1,3,5‐trimethylbenzene (TMB) were obtained from Shanghai Macklin Biochemical Co. Tetraethylorthosilicate (TEOS, 99.0%) was purchased from Sinopharm Chemical Reagent Co., Ltd. (Xi'an, China). Hexadecyl trimethyl ammonium bromide (CTAB) was purchased from Tianjin Kemiou Chemical Reagent Co., Ltd. (Tianjin, China). Dimethyl sulfoxide (DMSO), toluene, hydrochloric acid, and sodium hydroxide were purchased from Tianjin HengXing Chemical Reagent Co., Ltd. (Tianjin, China).

### Cell Culture

The experimental protocols were approved by the Hospital Ethics Committee (No. IRB‐REV‐2018020). Consent forms were obtained before conducting this research project. Healthy and periodontitis human tooth samples were collected from 12 individuals, 6 samples for each group, aged 20–40 years. Donor information is listed in Table S1 in the Supporting Information. Teeth were extracted at the Department of Oral and Maxillofacial Surgery, School of Stomatology, Fourth Military Medical University. PDLSCs were isolated as previously described.^[^ ^37^ ^]^ Briefly, to obtain single‐cell suspensions, periodontal ligament tissues were separated from the middle third of the root surface and digested in 3 mg mL^−1^ collagenase I (Sigma‐Aldrich, St. Louis., MO, USA) for 2 h at 37 °C. H‐BMMSCs were collected from the bone marrow aspirates of the iliac crest from healthy individuals, while A‐BMMSCs were obtained from knee specimens from osteoarthritis individuals who were undergoing total knee replacement surgery. Healthy bone marrow aspirates of the iliac crest from healthy individuals and knee specimens from osteoarthritis individuals were obtained from Xijing Hospital, Fourth Military Medical University. Healthy and osteoarthritis samples were collected from 12 individuals, with 6 samples for each group, aged 50–70 years. Donor information is listed in Table S2 in the Supporting Information. BMMSCs were purified using monocyte separation medium (Sigma‐Aldrich) as described previously.^[^ ^38^, ^39^ ^]^ Cells were then plated in six‐well culture dishes (Corning, NY, USA) and cultured in *α*‐minimal essential medium (*α*‐MEM; Gibco, USA) with 10% fetal bovine serum (Hangzhou Sijiqing Biological Engineering Materials Co., Ltd. Zhejiang, China), 0.292 mg mL^−1^ L‐glutamine (Invitrogen Life Technology, Carlsbad, CA, USA), 100 units mL^−1^ penicillin (Invitrogen), and 100 mg mL^−1^ streptomycin (Invitrogen) at 37 °C in 5% CO_2_. The medium was changed every 3 days. MSCs at 3–5 passages were used in this study. TNF‐*α* (10 ng mL^−1^, Sigma‐Aldrich) was added to the culture system at 10 ng mL^−1^ for 7 days to mimic the inflammatory microenvironment.

### Transmission Electron Microscopy

Cells were washed with serum‐free media and fixed with 4% glutaraldehyde and 4% paraformaldehyde (Sigma­Aldrich), dehydrated in a graded ethanol series and embedded in situ in LX‐812 resin (Ladd Research Industries Inc., Williston, VT, USA). Ultrathin sections were stained with uranyl acetate for 30 min and lead citrate for 10 min and observed by an FEI Tecnai G12 Spirit BioTwin transmission electron microscope (FEI Company, Hillsboro, Oregon, USA) with an accelerating voltage of 120 kV. To evaluate the frequency of contact between the mitochondria and the ER, images were taken at 43 000 × magnification. The perimeter of each mitochondrion was measured by ImageJ 1.52v (Media Cybernetics, USA) and the proportion of the mitochondrial surface closely associated with the ER was calculated.

### Organelle Fluorescence Staining

ER‐tracker and Mito‐Tracker double staining were used to evaluate the morphology of mitochondria and ER‐mitochondria colocalization in MSCs as previously reported.^[^ ^10^ ^]^ Briefly, MSCs were incubated in *α*‐MEM supplemented with 1 × 10^−3^ m ER‐Tracker green (Thermo Fisher Scientific, MA, USA) and 100 × 10^−9^ m Mito‐Tracker deep red (Thermo Fisher Scientific) at 37 °C for 30 min.

For the mitophagy assay, Lyso‐Tracker green (Yeasen Biotech Co., Ltd., Shanghai, China) and Mito‐Tracker deep red double staining were used to evaluate mitophagy. MSCs were incubated in *α*‐MEM supplemented with 50 × 10^−9^ m Lyso‐Tracker green and 100 × 10^−9^ m Mito‐Tracker deep red at 37 °C for 40 min. After washing, the medium was replaced with fresh *α*‐MEM. For the autophagy flux assay, MSCs were transfected with adenoviruses expressing mRFP‐GFP‐LC3 (GENECHEM, Shanghai, China) for 72 h. The nuclei were stained with 1 µg mL^−1^ Hoechst 33342 (Sigma‐Aldrich).

Live‐cell images were captured using a Nikon laser scanning confocal microscope (A1 Plus, Nikon, Japan). Thresholded images were evaluated using the “Analyze Particles” function in ImageJ (Media Cybernetics, USA) to obtain the number, perimeter, and area of mitochondrial fragments per cell. Quantification of colocalizations was performed with the Colocalization Plugin in ImageJ 1.52v.

### Detection of Mitochondrial Membrane Potential, ROS, and Mitochondrial Calcium

Mitochondrial membrane potential, ROS, and mitochondrial calcium in PDLSCs and BMMSCs were measured using a JC‐1 assay kit (Beyotime Institute of Biotechnology, Shanghai, China), ROS assay kit (Beyotime Institute of Biotechnology), and mitochondrial calcium concentration detection kit (Genmed Scientifics Inc., Wilmington, DE, USA), respectively, according to the manufacturer's instructions.

The parameters were measured with a multimode plate reader (HH3400, PerkinElmer, MA, USA). Mitochondrial membrane potential was determined by the fluorescence intensity ratio of J‐aggregates (Ex/Em = 525/590 nm) to monomers (Ex/Em = 490/530 nm). ROS were detected by the fluorescence intensity of DCF (Ex/Em = 488/525 nm).

The mitochondrial calcium concentration was measured by the fluorescence intensity of the mitochondrial calcium‐specific fluorescent probe rhod‐2‐AM (Ex/Em = 550/590 nm). Briefly, 96‐well plates were removed, and then maximum control wells, sample wells, and blank control wells were set. The relative fluorescence peak was measured by fluorescence spectrophotometry to determine the total calcium concentration in mitochondria using the following equation: intramitochondrial [Ca^2+^] = [(sample RFU – blank control RFU)/(maximum control RFU − sample RFU)] × 570 (× 10^−9^ m).

### Preparation of Mesoporous Silica Nanoparticles with Large Pores and CTAB Template (MSNT)

The preliminary MSNs were first fabricated by the classical CTAB‐templated, base‐catalyzed sol–gel method according to previous work.^[^ ^40^ ^]^ Briefly, the pH value of 1000 mL deionized water was adjusted to ≈11 with 52.8 mL ammonium hydroxide (25–28 wt% NH_3_·H_2_O). The temperature was raised to 323 K, and then 1.12 g CTAB was added. After the CTAB was completely dissolved, 5.8 mL TEOS was added dropwise with rapid stirring. After 2 h, the mixture was incubated overnight, centrifuged and washed thoroughly with distilled water and ethanol. The as‐synthesized preliminary MSNs were further dispersed in ethanol by sonication for 30 min, followed by the addition of 20 mL of a 1:1 mixture (v/v) of water and 1,3,5‐trimethylbenzene (TMB). The mixture was placed in the autoclave and kept at 140 °C for 4 days without stirring. The resulting white powder was washed with ethanol and water five times each and then dried under vacuum for 20 h.

### Preparation of TPP and Amino Comodified MSNT (MSNT‐TPP‐NH2)

A total of 2.62 g (1 eq, 10 mmol) of TPP and 1.68 g (1.1 eq, 11 mmol) of 3‐bromopropionic acid were dissolved in 20 mL of toluene and refluxed at 90 °C overnight. After cooling to room temperature, the suspension was centrifuged at 12 000 rpm for 5 min to collect the precipitate, which was washed with toluene three times to obtain carboxyl‐functionalized TPP (TPP‐COOH).

The resulting TPP‐COOH was dissolved in 20 mL of anhydrous tetrahydrofuran (THF), and the solution was refluxed overnight at 90 °C after adding 3.09 g (1.5 eq, 15 mmol) of *N,N*‐dicyclohexyl carbonate diimide (DCC), 100 mg of 4‐dimethylaminopyridine (DMAP), and 2.342 mL (1 eq, 10 mol) of 3‐aminopropyltriethoxysilane (APTES). After cooling to room temperature and removing the insoluble substance by centrifugation, the above solution was mixed with 180 mg of as‐synthesized MSNs‐T, 60 µL of APTES, and 10 mL of THF. The suspension was refluxed for 24 h at 90 °C. Due to the existence of the CTAB template in the pores, modification only occurred on the surface of MSNs, resulting in TPP and amino comodified MSN_T_ (MSN_T_‐TPP‐NH_2_). The nanoparticles were collected by centrifuging at 12 000 rpm for 5 min, washed with THF five times each, and then dried under vacuum for 20 h.

### Preparation of Template‐Removed MSNT‐TPP‐NH2 with a Graft of Trimethyl[3‐(trimethoxysilyl)propyl]ammonium Chloride in the Pores (TMA‐MSN‐TPP‐NH2)

The surfactant template of as‐synthesized MSN_T_‐TPP‐NH_2_ was removed by extraction using acidic methanol (9 mL of HCl/400 mL of methanol, 36 h) at 70 °C. After, the nanoparticles were centrifuged, washed several times with ethanol, and dried under vacuum for 20 h. The resulting MSN‐TPP‐NH_2_ was dispersed in THF containing 120 µL of trimethyl [3‐(trimethoxysilyl)propyl] ammonium chloride (TMA), followed by overnight stirring to generate nanoparticles with TMA in the pores (TMA‐MSN‐TPP‐NH_2_). The obtained nanoparticles were washed with THF and dried in a vacuum oven at 60 °C overnight.

### Preparation of the Final TMA‐MSN‐TPP‐EGTA‐PEG (METP) Nanoregulator

A total of 129.6 mg of EGTA and 54 mg of sodium hydroxide were dissolved in 10 mL of deionized water, and then 262 mg of 1‐(3‐dimethylaminopropyl)‐3‐ethylcarbodiimide (EDC) and 78 mg of *N*‐hydroxysuccinimide (NHS) were added. After adjusting the pH value of the solution to 5.0, 60 mg of TMA‐MSN‐TPP‐NH_2_ was added, followed by 10 min of sonication. This suspension was maintained at room temperature with stirring overnight. The obtained nanoparticles (TMA‐MSN‐TPP‐EGTA) were washed with deionized water and dried in a vacuum oven at 60 °C overnight. Subsequently, the as‐prepared TMA‐MSN‐TPP‐EGTA was redispersed in 10 mL of DMSO containing EDC (786 mg), DMAP (100 mg), and PEG_4000_ (5 g). The suspension was refluxed for 4 days at 60 °C. After the reaction, the suspension was cooled to room temperature, centrifuged at 12 000 rpm for 5 min, washed three times with deionized water, and then freeze‐dried.

### RNA Loading and pH/Esterase‐Triggered Release from METP

The loading of RNA (si*β*‐catenin) with and without a fluorescence label into METP NPs was achieved via electrostatic interactions. METP NPs (3 mg) and 25 nmol of RNA were mixed with 120 µL of nuclease‐free water at 4 °C for 2 h under gentle shaking. The mixture was further centrifuged and washed three times with nuclease­free water to obtain the RNA‐loaded METP NPs.

To investigate the pH‐ and esterase‐triggered RNA release, 0.5 mg of fluorescent RNA‐loaded METP NPs was dispersed in 1000 µL of nuclease­free water under different conditions (a: nuclease­free water at pH 7.4; b: nuclease­free water at pH 6.8; c: nuclease­free water at pH 4 and esterase; d: nuclease­free water at pH 7.4 and esterase; e: nuclease­free water at pH 4). Subsequently, the supernatant was obtained periodically from the suspension after centrifugation (12 000 rpm, 10 min). The amount of RNA released from the nanoparticles was determined by the fluorescence intensity of these supernatants at 530 nm, which was measured by a fluorescence spectrophotometer (Guangdong F­280, China).

### Animal Care

8 week old male C57BL/6 mice for the osteoarthritis model and 8 week old male SD rats for the periodontitis model were purchased from the Animal Center of the Fourth Military Medical University of China. All experimental procedures were approved by the Animal Protection Committee of the Fourth Military Medical University, China.

### Animal Models of Periodontitis and Osteoarthritis

8 week old C57BL/6 mice and 8 week old male SD rats were divided into 5 groups of 6 samples each. One group was set as a control group (for periodontitis) or sham group (for OA), and four groups were treated as disease models and treatment groups: TMA‐MSNs‐TPP, METP NPs, TMA‐MSNs‐TPP/si*β*‐catenin, and METP NPs/si*β*‐catenin.

Experimental periodontitis was induced by LPS injection as previously reported.^[^ ^41^ ^]^ Briefly, SD rats were anesthetized, and 10 µL saline or LPS (1 mg mL^−1^, Sigma‐Aldrich) was injected in each group into the right maxillary palatal gingiva between the first and second upper molars and repeated every other day for 7 days. Then, 20 µL saline, TMA‐MSNs‐TPP (4 mg mL^−1^), METP NPs (4 mg mL^−1^), TMA‐MSNs‐TPP/si*β*‐catenin (4 mg mL^−1^), and METP NPs/si*β*‐catenin (4 mg mL^−1^) were injected into the same site weekly in each group. All rats were anesthetized and sacrificed by exsanguination 28 days after NP treatment.

To establish osteoarthritis models, C57BL/6 mice were anesthetized and then the ACL and the medial meniscus were partially transfected to induce mechanical instability‐associated osteoarthritis in the right knee as previously described.^[^ ^42^ ^]^ In the treated group, 20 µL of NPs was injected into subchondral bone weekly at 30 days after ACLT+MMx surgery. For the sham group, the same amount of saline was injected into the same site as the treated group. Mice were euthanized 30 days after NP treatment.

### Micro‐CT Analysis

To evaluate the level of periodontal recession, the maxillary jaws were scanned and analyzed using a micro‐CT system (Siemens Inveon Micro‐CT, Munich, Germany). After the maxillary jaws were scanned, rebuilt images were used to perform 3D histomorphometric analysis. The alveolar bone height was measured at four different sites in two molars by recording the distance from the CEJ to the alveolar bone crest.

Right knee joints were dissected from mice free of soft tissue and analyzed by high‐resolution micro‐CT. 3D models were reconstructed to analyze parameters of the tibial subchondral bone. Structural parameters analyzed included TV (total tissue volume; contained both trabecular and cortical bone), SBP. Th (subchondral bone plate thickness), and Tb. Pf (trabecular pattern factor).

### Histomorphometry Staining

At the time of euthanasia, the maxillary jaws of periodontitis rats and the right knee joints of OA mice were resected and fixed in 10% buffered formalin for 48 h, decalcified in 17% EDTA (pH 7.4) for 30 days, and embedded in paraffin. 3 *μ*m thick cross‐sections of the periodontal tissue were processed for H&E staining. 3 *μ*m thick sagittal‐oriented sections of the knee joint medial compartment were processed for safranin O (Sigma‐Aldrich) and fast green (Sigma‐Aldrich) staining according to the manufacturer's instructions. Images were obtained with an Olympus BX41 Microscope (Olympus, Japan). OARSI scores were calculated (score = grade × stage) as previously described.^[^ ^22^ ^]^ The score represented the maximum of the tibial scores at the medial and lateral side. The articular cartilage area on OA mouse sections was evaluated using ImageJ.

### Statistical Analysis

All experiments were repeated at least three times, and the data were presented as the mean ± SD. Statistical analysis was performed by Student's *t* test (two­tailed), one­way analysis of variance (ANOVA) or Kruskal–Wallis *H* test with GraphPad Prism 8.0 (GraphPad Software, USA). Tukey's post hoc test was used for multiple post hoc comparisons to determine the significance between the groups after one­way ANOVA. Dunn's multiple comparison test was used for multiple post hoc comparisons after the Kruskal–Wallis *H* test. The difference between groups was considered statistically significant for *p < 0.05, very significant for **p < 0.01, and the most significant for ***p < 0.001. Graph analysis was performed using GraphPad Prism 8.0 (GraphPad Software, USA).

## Conflict of Interest

The authors declare no conflict of interest.

## Author Contributions

Q.Z., X.C., D.F., and X.G. contributed equally to this work. Q.Z., X.G., and D.F. contributed to study design, execution, data acquisition, and interpretation; X.H. and Z.W. performed the animal experiments; S.S. contributed to study design and interpretation; F.J., X.C., and Y.J. oversaw the collection of results and data interpretation, and drafted the reports. B.L. designed the experiments, oversaw the collection of results and data interpretation, and drafted the reports. All authors have seen and approved the final version.

## Supporting information

Supporting Information

## Data Availability

Research data are not shared.
