# Supplementary material for: Nanorepairers Rescue Inflammation‐Induced Mitochondrial Dysfunction in Mesenchymal Stem Cells
Source: Adv Sci (Weinh). 2021 Dec 11;9(4):2103839. doi: 10.1002/advs.202103839 (PMC8811813; doi:10.1002/advs.202103839)
Supplement: Supplementary file 1 — Supporting Information [file ADVS-9-2103839-s001.pdf]

ADVANCED SCIENCE
Open Access

Supporting Information

for *Adv. Sci.*, DOI: 10.1002/advs.202103839

Nano-repairers rescue inflammation-induced mitochondrial dysfunction in mesenchymal stem cells

Qiming Zhai, Xin Chen, Dongdong Fei, Xiaoyan Guo, Xiaoning He, Wanmin Zhao, Songtao Shi, John Justin Gooding, Fang Jin*, Yan Jin*, Bei Li**

Supporting Information

Nano-repairers rescue inflammation-induced mitochondrial dysfunction in mesenchymal stem cells

Qiming Zhai, Xin Chen, Dongdong Fei, Xiaoyan Guo, Xiaoning He, Wanmin Zhao, Songtao Shi, John Justin Gooding, Fang Jin*, Yan Jin*, Bei Li**

This file includes:

Figures S1 to S8
Tables S1 to S3
Supplementary Materials and Methods
Reference [1]

Supplementary Figures

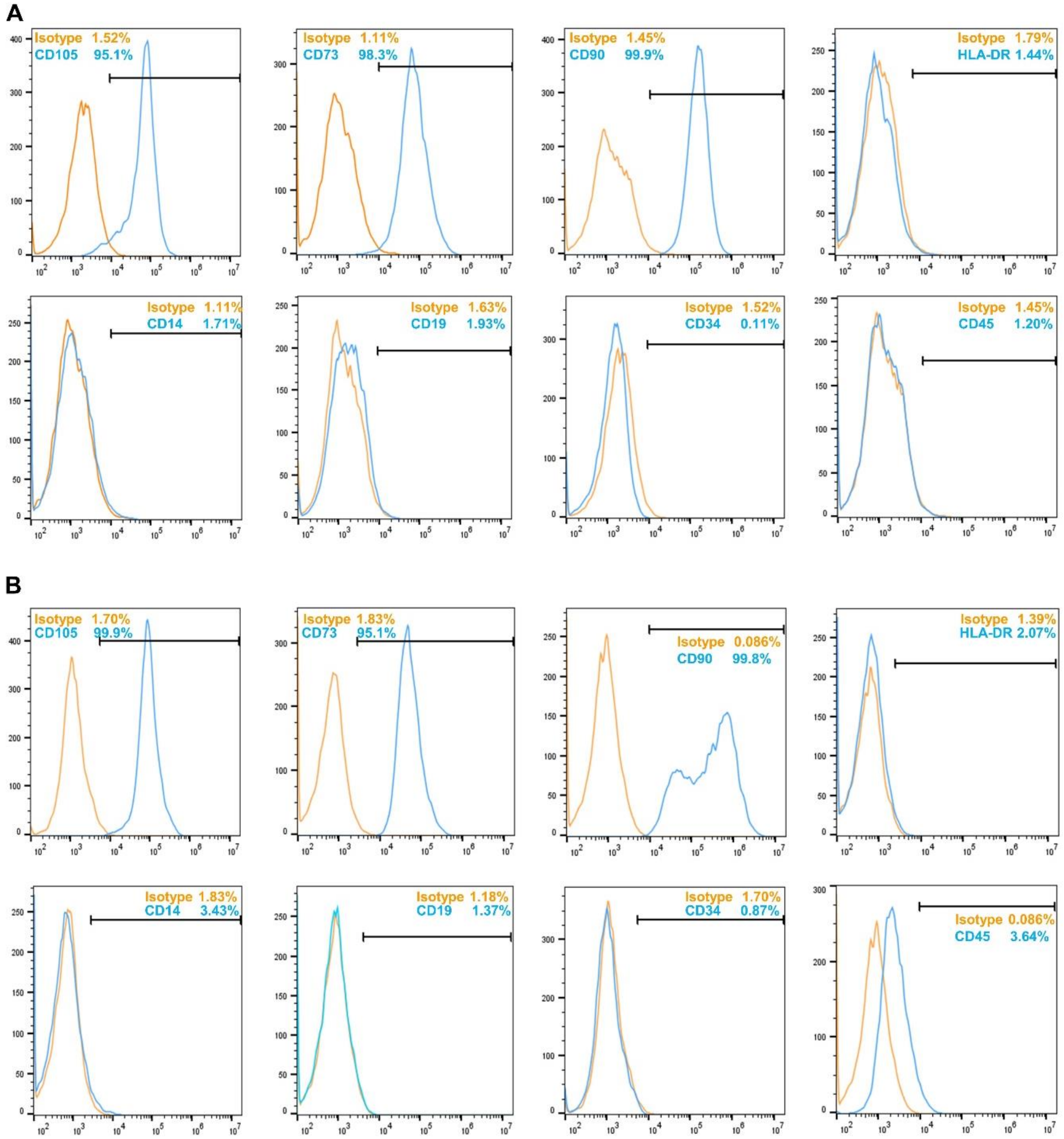

Figure S1. Flow cytometric analysis of PDLSCs and BMMSCs. Both (A) PDLSCs and (B) BMMSCs populations reveal positive expression of CD105, CD90, CD73, and negative expression of CD45, CD34, CD14, CD19, and HLA-DR.

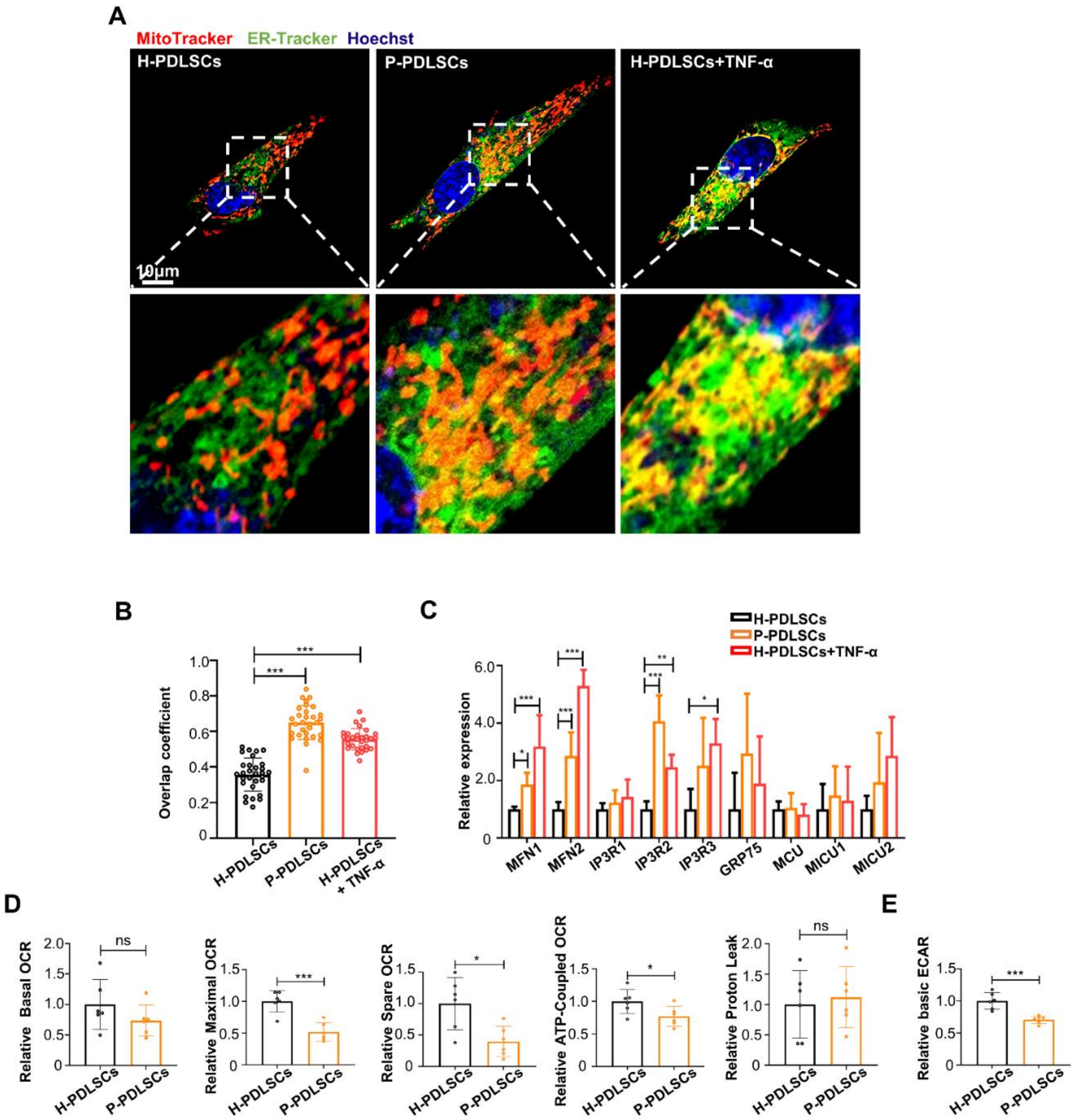

Figure S2. Mitochondrial Ca^{2+} overload results in dysfunctional mitochondria in MSCs derived from periodontitis patients. (A) Representative images of H-PDLSCs, P-PDLSCs and H-PDLSCs+TNF-α co-expressing a MitoTracker (Red) and ER-Tracker (Green). Scale bar, 10μm. **(B)** Statistical quantification of the overlap coefficient between MitoTracker (Red) and ER-Tracker (Green) (*n* = 30 cells in each group). **(C)** The expression of MFN1, MFN2, IP3R1, IP3R2, IP3R3, GRP75, MCU, MICU1 and MICU2 in H-PDLSCs, P-PDLSCs and H-PDLSCs+TNF-α were measured by qRT-PCR (*n* = 3-6 independent samples in each group). (D) Calculated relative basal, maximal, spare, proton leak and ATP-coupled OCR in H-PDSLCs and P-PDSLCs (*n* = 6 independent samples in each group). (E) Calculated relative basal ECAR in H-PDSLCs and P-PDSLCs (*n* = 6 independent samples in each group). *P < 0.05. **P < 0.01, ***P < 0.001.

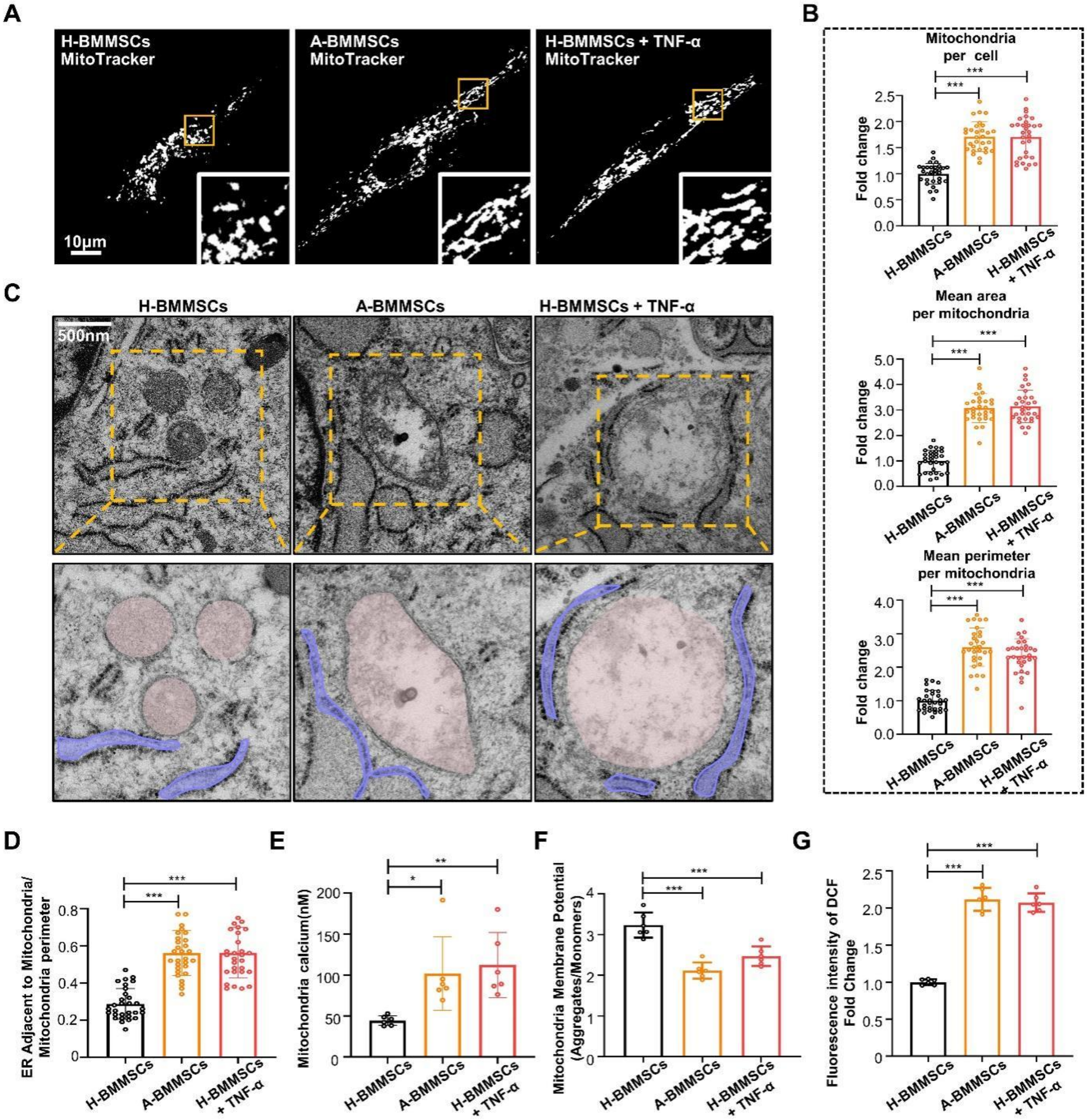

Figure S3. Mitochondrial Ca^{2+} overload results in dysfunctional mitochondria in MSCs derived from osteoarthritis patients. (A) Representative confocal images of H-BMMSCs, A-BMMSCs and H-BMMSCs+TNF-α expressing MitoTracker. Scale bar, 10μm. **(B)** Respective mitochondrial morphology analysis of cells by number of mitochondria per cell, and mean area and perimeter per mitochondrion (*n* = 30 cells in each group). **(C)** Representative transmission electron microscopy (TEM) images of H-BMMSCs, A-BMMSCs and H-BMMSCs+TNF-α at 43000x, scale bar 500nm. Structures colored by purple indicate endoplasmic reticulum; Structures colored by pink indicate mitochondria. **(D)** Quantitation of ER length adjacent to mitochondria normalized by total mitochondrial perimeter (*n* = 30 cells in each group). **(E)** Mitochondrial calcium was detected in H-BMMSCs, A-BMMSCs and H-BMMSCs+TNF-α by Rhod-2

(*n* = 6 independent samples). **(F)** Mitochondrial membrane potential in H-BMMSCs, A-BMMSCs and H-BMMSCs+TNF-α analyzed by JC-1 assay (*n* = 6 independent experiments). **(G)** ROS in H-BMMSCs, A-BMMSCs and H-BMMSCs+TNF-α detected by DCFH-DA assay (*n* = 6 independent experiments). *P < 0.05. **P < 0.01, ***P < 0.001.

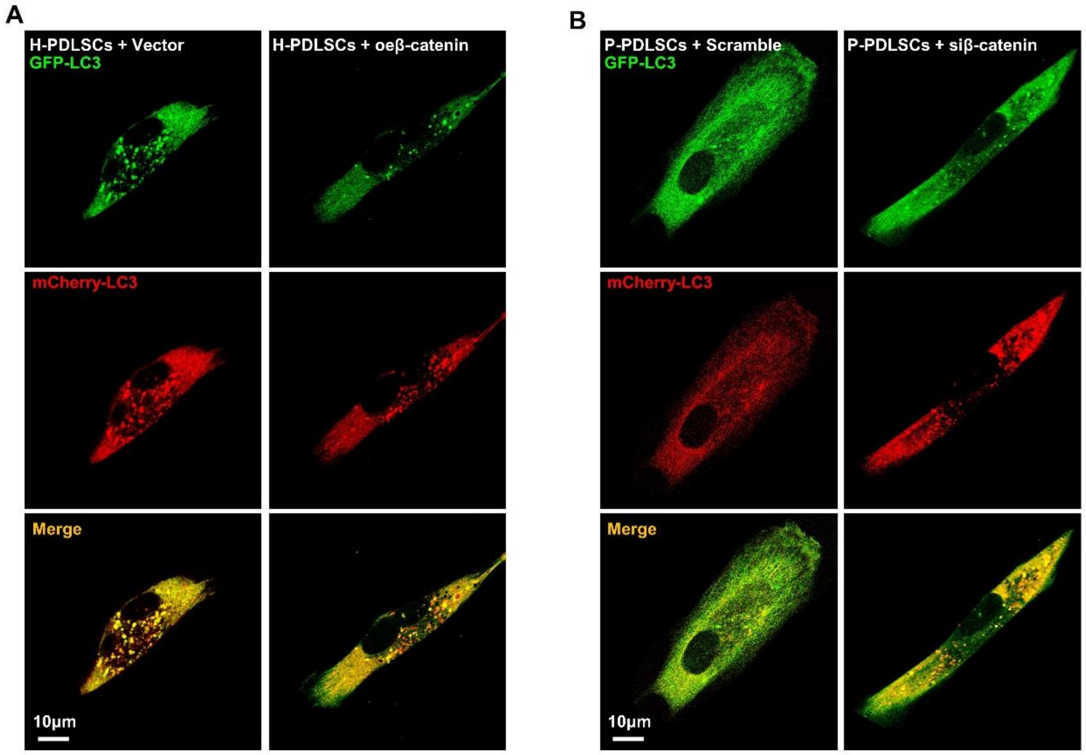

Figure S4. β-Catenin suppresses autophagosome formation in PDLSCs. Representative confocal microscopy images of autophagosomes and autolysosomes detected by adenoviruses expressing mRFP-GFP-LC3 **(A)** in H-PDSLCs with β-catenin overexpression and (**B**) in P-PDSLCs with β-catenin knockdown. Scale bar, 10 μm.

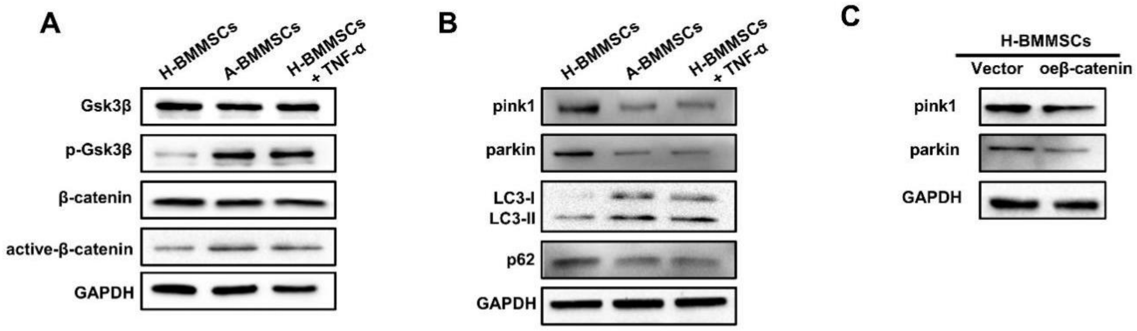

Figure S5. Mitophagy is inhibited by Wnt/β-catenin pathway in MSCs derived from osteoarthritis patients. (A) Activated Wnt/β-catenin pathway in A-BMMSCs and H-BMMSCs+TNF-α was detected by western blot assay. **(B)** The expression of pink1, parkin, LC3 Ⅰ, LC3 Ⅱ and p62 in H-BMMSCs, A-BMMSCs and H-BMMSCs+TNF-α were detected by western blot assay. **(C)** The expression of pink1 and parkin were suppressed after β-catenin overexpression in H-BMMSCs.

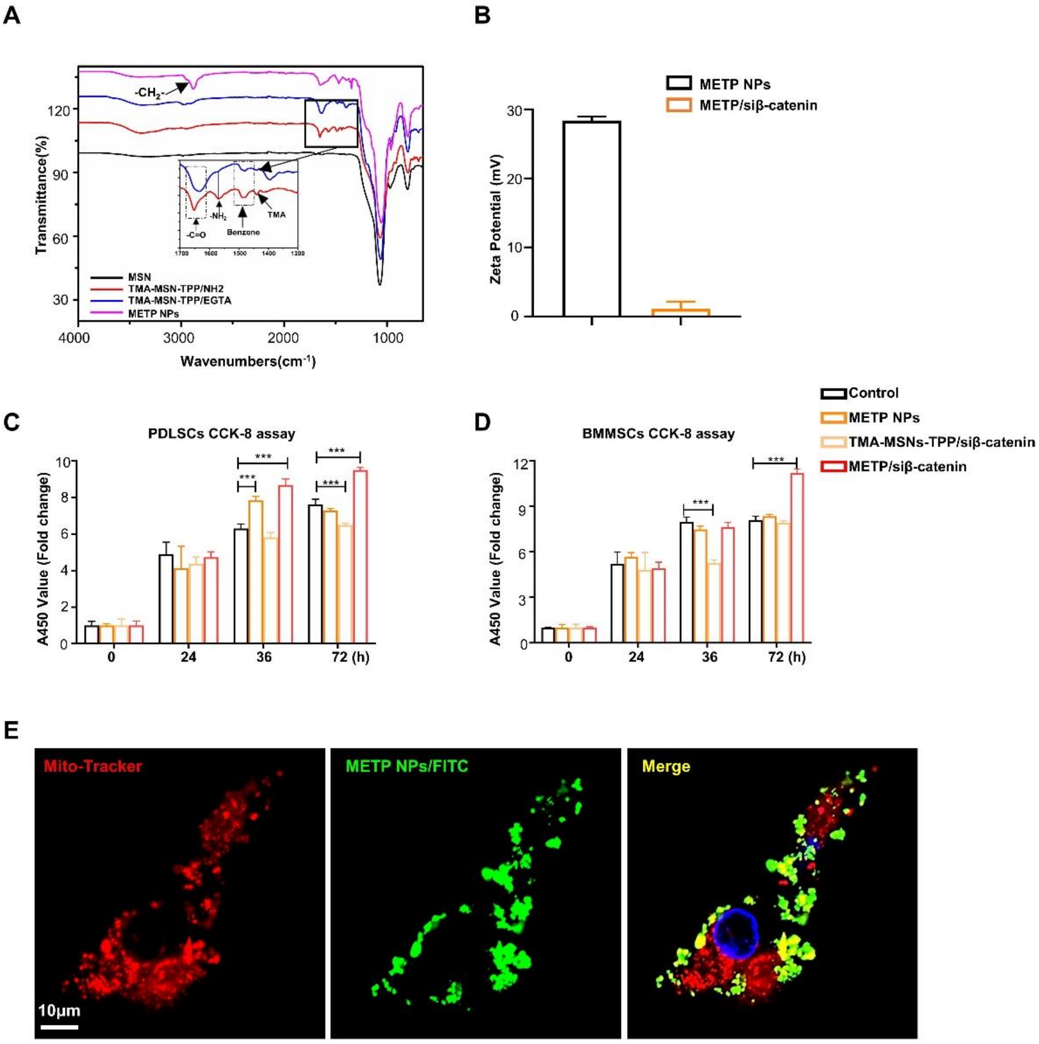

Figure S6. Characteristics of METP NPs. (A) Infrared spectrum of TMA-MSN-TPP/EGTA-PEG nanoparticles (METP NPs) as well as the intermediate products during its fabrication (MSN, TMA-MSN-TPP/NH2 and TMA-MSN-TPP/EGTA), which indicated the stepwise formation of METP NPs. **(B)** Zeta potential measurement of METP NPs before and after RNA loading. **(C, D)** Cell viability of (C) PDLSCs and (D) BMMSCs after treated by METP NPs, siβ-catenin loaded TMA-MSNs-TPP (TMA-MSNs-TPP/siβ-catenin) and siβ-catenin loaded METP NPs (METP/siβ-catenin), which were determined by CCK-8 assay kit (*n* = 3 independent experiments. **(E)** Confocal fluorescence microscope images of MSCs after incubation with FITC labelled METP NPs, in which the mitochondria were stained by MitoTracker (Red), indicating the mitochondrial targeting ability of the METP NPs. Scale bar: 10 μm. *P < 0.05, **P < 0.01, ***P < 0.001.

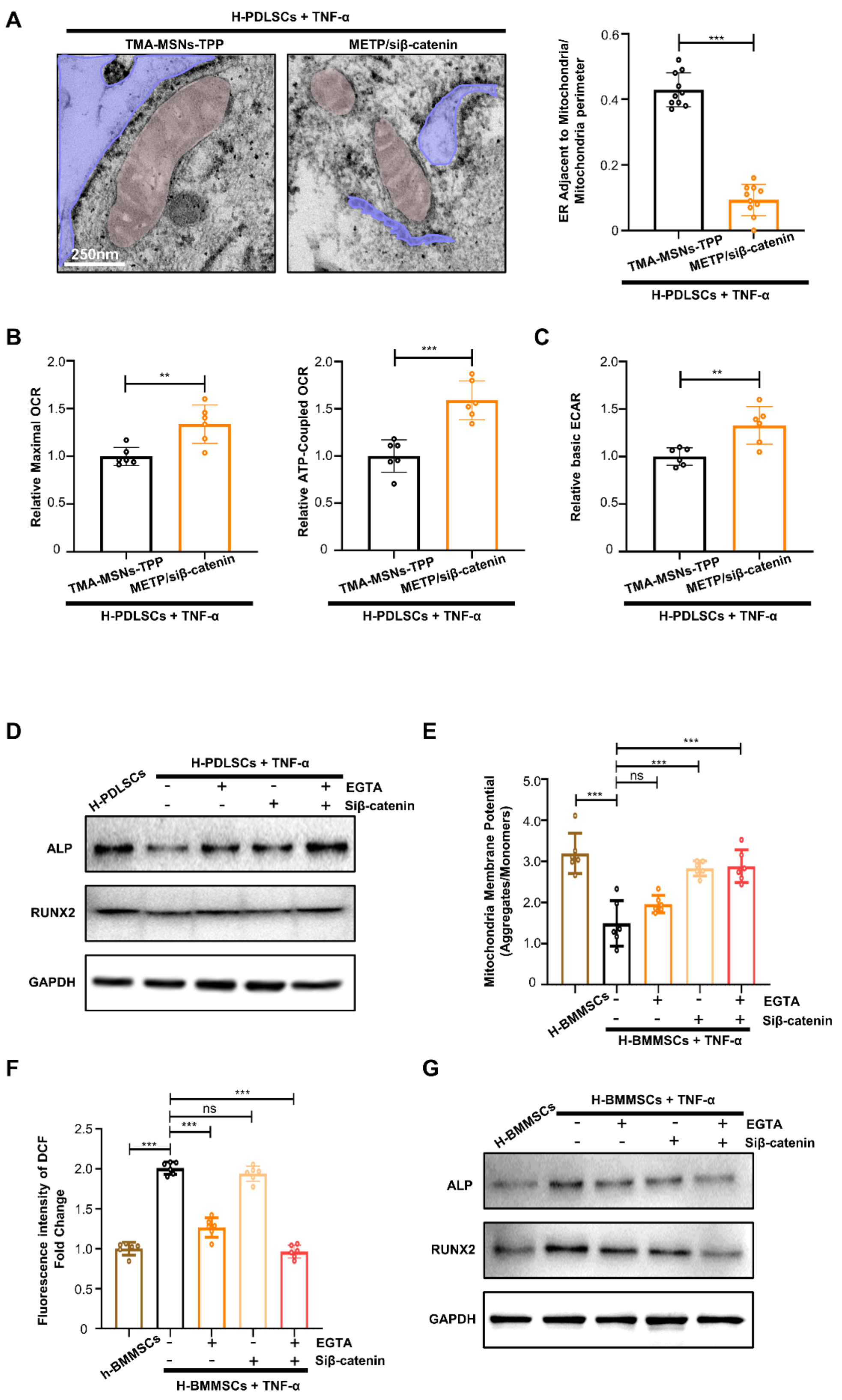

Figure S7. The rescue effect of METP/siβ-catenin in PDLSCs and BMMSCs. (A) Representative transmission electron microscopy (TEM) images of H-PDLSCs+TNF-α with treatment of TMA-MSNs-TPP and METP/siβ-catenin. Scale bar, 250nm. **(B)** Calculated relative maximal and ATP-coupled OCR in

H-PDLSCs+TNF-α with treatment of TMA-MSNs-TPP and METP/siβ-catenin. **(C)** Calculated relative basal ECAR in H-PDLSCs+TNF-α with treatment of TMA-MSNs-TPP and METP/siβ-catenin. **(D)** The expression of osteogenesis associated protein ALP and RUNX2 in PDLSCs after different NPs treatment (*n* = 6 independent experiments). **(E)** Recovery of mitochondria membrane potential in BMMSCs after treated by TNF-α with different NPs (*n* = 6 independent experiments). **(F)** Recovery of ROS levels in BMMSCs after treated by TNF-α with different NPs (*n* = 6 independent experiments). **(G)** The expression of osteogenesis associated protein ALP and RUNX2 in BMMSCs after different NPs treatment (*n* = 6 independent experiments). ***P < 0.001, ns, not significant.

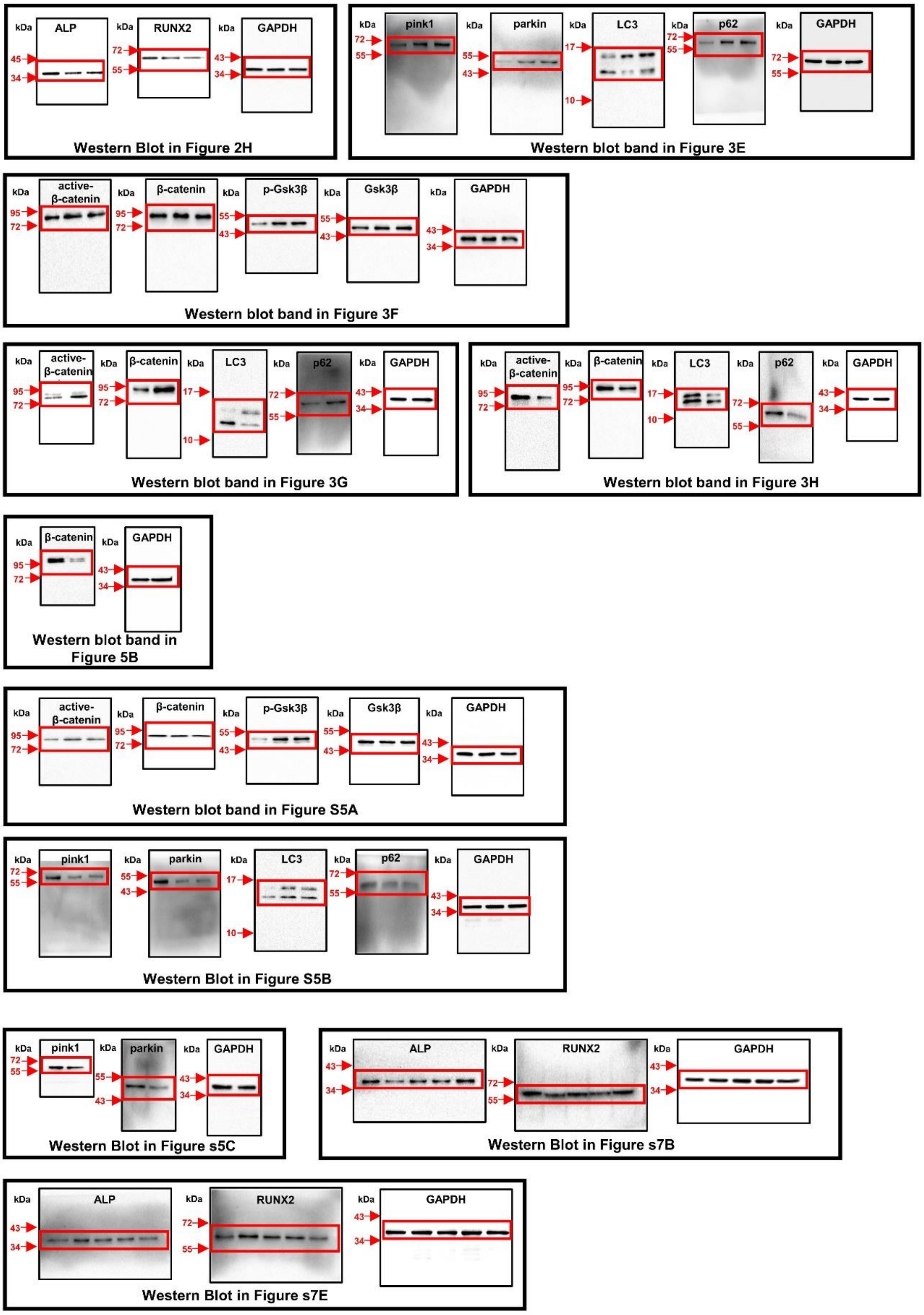

Figure S8. Uncropped images of the western blot bands

Supplementary Tables

Table S1. Donor information of human PDSLCs.

Cells	Age	Gender
H-PDLSCs1	20	Male
H-PDLSCs2	27	Female
H-PDLSCs3	33	Male
H-PDLSCs4	39	Female
H-PDLSCs5	29	Male
H-PDLSCs6	33	Female
P-PDLSCs1	20	Male
P-PDLSCs2	28	Female
P-PDLSCs3	27	Male
P-PDLSCs4	40	Female
P-PDLSCs5	39	Male
P-PDLSCs6	37	Male

Table S2. Donor information of human BMMSCs.

Cells	Age	Gender
H-BMMSCs1	52	Female
H-BMMSCs2	62	Male
H-BMMSCs3	54	Female
H-BMMSCs4	61	Male
H-BMMSCs5	60	Female
H-BMMSCs6	63	Male
A-BMMSCs1	55	Female
A-BMMSCs2	63	Male
A-BMMSCs3	57	Female
A-BMMSCs4	60	Male
A-BMMSCs5	65	Female
A-BMMSCs6	69	Male

Table S3. Sequence of primers for qRT-PCR.

mRNA/primer	Forward (5'-3')	Reverse (5'-3')
h-GAPDH	GCACCGTCAAGGCTGAGAAC	TGGTGAAGACGCCAGTGGA
h-MFN1	TTGAGAGATGACCTGGTGTTAGTAG	ATTGAGAATGAAAATATTAGGCTTG
h-MFN2	TCAAGACTATAAGCTGCGAATT	GAGGACTACTGGAGAAGGGTG
h-IP3R1	ATGTTCGGCTCAGACACCTAT	TGCTTCCTTATCCTCCTTCAC
h-IP3R2	AGAGGACAATGACAATGCGGAGAC	CCAGACAGCAAAGGCGGGTAGT
h-IP3R3	TCAGAACAACAAAACCAACTACAAC	TCGTGAGTCACAATGCAAGTCT
h-MCU	CACTGTTGTGCCCTCTGATGAT	AACTCTGTCAATTCCCCGATC
h-MICU1	TGTGATGGCAATGGCGAACTGA	CTGCATGAGGCGAGTGAAACCC
h-MICU2	TATGACACCACGAGACTTCCTC	CATTACCATCTGTATCCAGCATTT
h-GRP75	AGAAAGGAGTATTTGAGGTGA	AGAATCCATTGTAAGATAGGG

Supplementary Materials and Methods

Flow cytometric analysis

Surface markers of MSCs were evaluated according to ISCT criteria. [1] PDLSCs and BMMSCs at passages 3-5 were analyzed for isotype control (FITC) (BioLegend, CA, USA), isotype control (PE) (BioLegend), CD14 (FITC) (eBioscience, San Diego, CA, USA), CD34 (PE) (BioLegend), CD45 (BioLegend), CD73 (FITC) (BioLegend), CD90 (PE) (eBioscience), CD19 (APC) (eBioscience), HLA-DR (FITC) (eBioscience) and CD105 (PE) (BioLegend) by flow cytometry. After digestion by trypsin, 1×10^6 cells were counted and incubated with the abovementioned antibodies at room temperature in the dark for 30 min. After washing with PBS twice, MSCs were subjected to flow cytometric analysis (Beckman Coulter, Fullerton, CA, USA).

Quantitative RT–PCR

Total RNA was extracted from macrophages by using TRIzol reagent (Invitrogen Life Technology, Carlsbad, CA, USA), and complementary DNA (cDNA) was synthesized with a PrimeScript RT reagent kit (TaKaRa, Japan) according to the manufacturer’s protocol. Real-time RT-PCR was performed in a 10 µl reaction volume using the SYBR Premix Ex Taq II kit (TaKaRa) and detected on the CFX96 Real-Time System (Bio-Rad, USA). The $2^{-\triangle\triangle Ct}$ method was used to calculate fold changes of mRNA. GAPDH was used as the internal control. The primer set sequences applied in this study are listed in Table S3.

Western blot analysis

The protein samples from cells were loaded into the Bio-Rad Electrophoresis System. The proteins in the gel were transferred to polyvinylidene difluoride (PVDF) membranes. After blocking in 5% bovine serum albumin (BSA) solution, the membranes were incubated at 4 °C overnight with the following primary antibodies: anti-LC3B antibody (Cell Signaling Technology, MA, USA), anti-p62 antibody (Cell Signaling), anti-parkin antibody (Cell Signaling), anti-pink1 antibody (Cell Signaling), anti-ALP antibody (R&D Systems, Minneapolis, MN, USA), anti-RUNX2 antibody (Cell Signaling), anti-GSK-3β (Cell Signaling), anti-phospho-GSK-3β antibody (Cell Signaling), anti-β-catenin antibody (Cell Signaling), and anti-active-β-catenin antibody (Cell Signaling). Then, the membranes were incubated with the corresponding secondary antibodies at room temperature for 1 hour. Membranes were visualized using an enhanced chemiluminescence kit (Amersham Biosciences, Piscataway, NJ, USA). and photographed using an imaging system (Tanon 5500, Shanghai).

Osteogenic differentiation

Before osteogenic induction, 2 × 10^5 PDLSCs and BMMSCs were seeded on twelve-well culture dishes and cultured in growth medium until the cells reached 60%–70% confluence as defined by the area covered. Then, the cells were cultured under osteogenic culture conditions consisting of 100 μg/ml ascorbic acid (Sigma-Aldrich), 5 mmol/l β- glycerophosphate (Sigma-Aldrich), and 10 nmol/l dexamethasone (Sigma-Aldrich). Osteogenesis-related proteins alkaline phosphatase (ALP) and runt-related transcription factor 2 (Runx2) were assayed by western blot after osteogenic induction for 14 days. Alizarin red staining was used to assess calcium deposits on day 28, and 10% cetylpyridinium chloride was added for quantitative analysis. The absorbance values were measured at 562 nm with a spectrophotometer (Epoch, BioTek, USA).

Transfection assays

For transfection with small interfering RNA, MSCs (5 × 10^5) were seeded on a 6-well culture plate and transfected with MFN1 siRNA, MFN2 siRNA (Santa Cruz, Dallas, Texas, USA) or β-catenin siRNA (GeneChem, Shanghai, China) using X-treme GENE siRNA Transfection Reagent (Roche, Basel, Switzerland) according to the manufacturer's instructions. For overexpression plasmid transfection of MFN1, MFN2, or β-catenin (GeneChem), we used the protocol of the X-treme GENE HP DNA Transfection Reagent (Roche). After transfection, cells were used for protein extraction for western immunoblotting or for confocal images.

Examination of relative Oxygen consumption rate (OCR) and extracellular acidification rate (ECAR)

Cells were seeded on 96 well plates. OCR and ECAR were detected using the MitoXpress oxygen-sensitive probe (Luxcel Biosciences, Cork, Ireland) and pHXtra pH-sensitive probe (Luxcel Biosciences, Cork, Ireland) according to the manufacturer's protocols. Time-resolved fluorescence measurements were performed with a fluorescence microplate reader (CLARIOstar, BMG LABTECH, Germany). For OCR evaluation, 1 μM oligomycin, 1 μM FCCP, and 1μM Antimycin A were added respectively to calculate the basal OCR (Untreated-Antimycin A), ATP-coupled OCR (Untreated-Oligomycin), maximal OCR (FCCP-Antimycin A), spare OCR (FCCP-Untreated) and proton leak (Oligomycin-Antimycin A). The data was expressed as relative experimental values.

Cytotoxicity assay

CCK-8 (Cell Counting Kit-8) for quantitation of viable cell number was used to examine the cytotoxicity of NPs in vitro. Briefly, PDLSCs and BMMSCs were seeded on ninety-six-well plates at a concentration of 10^4 cells per well. After cell adhesion, METP NPs (40 μg/ml), TMA-MSNs-TPP/siβ-catenin (40 μg/ml), and METP/siβ-catenin (40 μg/ml) were added to the plate. After 0, 24, 36, and 78 hours of treatment, cell viability was detected by the standard CCK-8 assay according to the instructions. The absorbance values at 450 nm were measured with a spectrophotometer (Epoch). The fold change in cell number was calculated by normalizing to the A450 value of the control group at 0 h.

Reference

[1] M. Dominici, K. Le Blanc, I. Mueller, I. Slaper-Cortenbach, F. C. Marini, D. S. Krause, R. J. Deans, A. Keating, D. J. Prockop, E. M. Horwitz, CYTOTHERAPY **2006**, 8, 315.
